# An Engineered Intravitreal Injection Retinal-Pigment-Epithelium-Tropic Adeno-Associated Virus Vector Expressing a Bispecific Antibody Binding VEGF-A and ANG-2 Rescues Neovascular Age-Related Macular Degeneration in Animal Models and Patients

**DOI:** 10.34133/research.0717

**Published:** 2025-05-29

**Authors:** Yuan Cai, Yonghao Gu, Jie Zhang, Ying Zhu, Zhen Ma, Qin He, Yongjia Sun, Mengmeng Yuan, Xiaojun Li, Kai Zhu, Bolong Miao, Jin Zhao, Juan Liu, Min Tang, Dali Tong, Lixia Feng, Ming Ma, Guisheng Zhong, Zilong Qiu, Tian Xue

**Affiliations:** ^1^ First Affiliated Hospital of USTC, School of Life Sciences, Division of Life Sciences and Medicine, University of Science and Technology of China, Hefei 230026, China.; ^2^ Starrygene Therapeutics Company Limited, Hefei 230031, China.; ^3^ Hefei National Research Center for Physical Sciences at the Microscale, Neurodegenerative Disorder Research Center, CAS Key Laboratory of Brain Function and Disease, University of Science and Technology of China, Hefei 230026, China.; ^4^ The First Affiliated Hospital of Anhui Medical University, Hefei 230032, China.; ^5^ iHuman Institute, ShanghaiTech University, Shanghai 201210, China.; ^6^ School of Life Science and Technology, ShanghaiTech University, Shanghai 201210, China.; ^7^ Songjiang Research Institute, Songjiang District Central Hospital, Shanghai Jiao Tong University School of Medicine, Shanghai 200025, China.; ^8^ Clinic Neuroscience Center, Department of Neurology, Ruijin Hospital, Shanghai Jiao Tong University School of Medicine, Shanghai 200025, China.

## Abstract

Antiangiogenesis gene therapy based on adeno-associated virus (AAV) vectors represents a promising advancement in the treatment of neovascular age-related macular degeneration (nAMD), providing an alternative to antibody-based therapies. However, the development of a safe and effective AAV vector capable of precisely targeting neovascularization and choroidal leakage remains a critical unmet need. In the present study, we engineered a novel intravitreally administered AAV vector with retinal-pigment-epithelium (RPE)-specific tropism. This vector demonstrated robust and localized gene expression in RPE cells while maintaining a favorable safety profile. The RPE-tropic AAV vector delivered a dual-acting antibody against vascular endothelial growth factor (VEGF) and angiopoietin-2 (ANG-2), exhibiting strong therapeutic efficacy and tolerability in both rodent and nonhuman primate choroidal neovascularization models. Based on the promising preclinical data, a single-center, single-arm, investigator-initiated trial (ChiCTR2400085329) was conducted to assess its safety and efficacy in patients with nAMD. The RPE-tropic AAV vector expressing anti-VEGF-A and anti-ANG-2 effectively alleviated disease progression and was well tolerated in the clinical setting. These findings highlight the potential of this engineered AAV-RPE capsid as a versatile platform for gene therapy, not only for nAMD but also for other ocular diseases involving RPE cells.

## Introduction

Neovascular age-related macular degeneration (nAMD) is a progressive retinal degenerative disease characterized by choroidal neovascularization (CNV), which drives subretinal hemorrhage and exudation, ultimately resulting in vision loss. As a leading cause of blindness worldwide, nAMD poses a substantial public health burden due to the irreversible nature of its associated vision impairment [1–4]. Vascular endothelial growth factor (VEGF) and angiopoietin-2 (ANG-2) are key molecular regulators of angiogenesis and play a central role in nAMD pathogenesis [5]. As such, these molecules are attractive targets for nAMD treatment. Current standard-of-care treatments for nAMD primarily involve anti-VEGF agents, with the recent addition of a bispecific antibody targeting both VEGF and ANG-2, which has shown promise in improving therapeutic outcomes [6,7]. Despite their efficacy, these therapies require frequent intravitreal (IVT) injections and regular clinic visits to sustain their benefits, presenting marked challenges for long-term patient compliance and real-world effectiveness [8–10]. As such, emerging therapeutic strategies for nAMD need to be further studied.

Adeno-associated virus (AAV) gene therapy offers a transformative approach to the treatment for ocular disorders by enabling long-term and stable expression of therapeutic genes. Unlike conventional antibody therapies, AAV-based treatments promise sustained drug delivery and long-term efficacy through a single injection [11], addressing important limitations of current therapeutic options. AAV gene therapy has also demonstrated a strong safety profile in ocular applications, such as Food and Drug Administration-approved Luxturna for inherited retinal diseases [12]. As a result, AAV vectors have emerged as a highly promising platform for treating nAMD, and several AAV-based therapies are currently under clinical evaluation for nAMD [13–16]. For example, ABBV-RGX-314, employing an AAV8 vector to express an anti-VEGF-A antigen-binding fragment in retinal pigment epithelial (RPE) cells, has reached phase III clinical trials. However, its administration via subretinal and suprachoroidal injection is associated with risks such as retinal detachment, tears, and macular holes [17]. In contrast, IVT injection offers a safer and more convenient alternative. ADVM-022, utilizing an AAV2.7m8 vector to deliver aflibercept, is administered through IVT treatment but primarily targets the inner nuclear layer, inner plexiform layer, and ganglion cell layer [18]. Similarly, 4D-150, which combines an R100 vector with a transgene payload encoding aflibercept and VEGF-C interference RNA, is administered via IVT injection but its expression spans multiple retinal layers without specificity to RPE cells [19]. The lack of RPE-specific tropism in current intravitreally administered AAV capsids may limit their therapeutic efficacy, underscoring the need for safer and more targeted gene therapy solutions for nAMD.

In this study, we developed XMVA09, an innovative and effective gene therapy candidate encoding a bispecific antibody that inhibits both VEGF-A and ANG-2. Notably, this novel AAV capsid achieved stable expression of bispecific antibodies specifically in RPE cells following IVT administration. Preclinical models demonstrated that XMVA09 effectively suppressed neovascularization and vascular leakage in CNV models in both mice and nonhuman primates (NHPs). NHP studies revealed that a single IVT injection of XMVA09 caused no therapy-related ocular inflammation or adverse changes in retinal function and morphology. Furthermore, building on these promising preclinical findings, we conducted an investigator-initiated clinical trial, which demonstrated that XMVA09 is well tolerated and delivers substantial clinical benefits in nAMD patients. Collectively, these results establish XMVA09 as a highly effective gene therapy for the treatment of nAMD. Furthermore, the novel RPE-tropic AAV capsid developed in this study holds considerable potential for broader applications in gene therapies targeting other ocular diseases.

## Results

### The novel RPE-tropic AAV capsid administered by IVT injection efficiently targets RPE cells in mice and NHPs

**Fig. 1.**
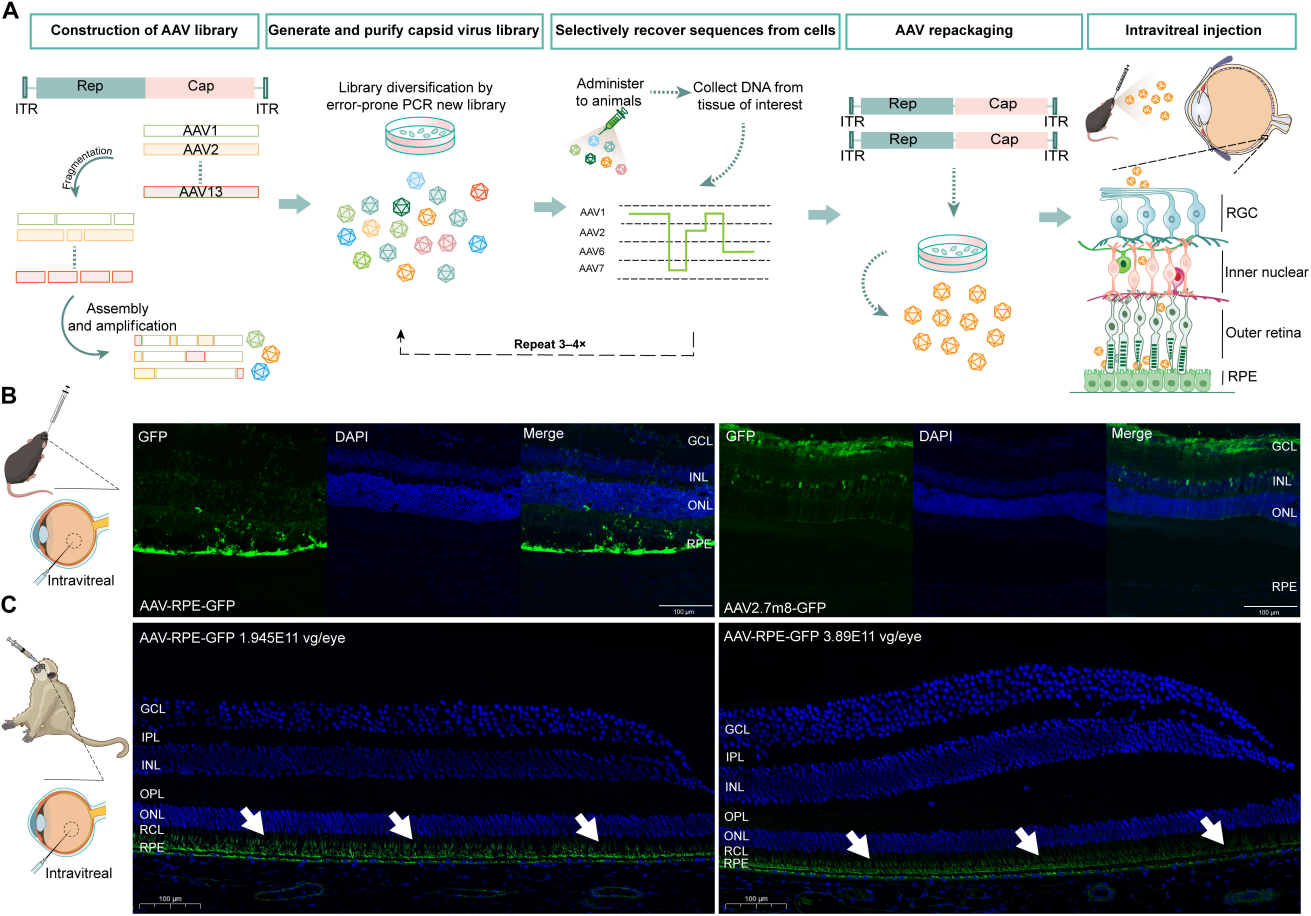
The novel AAV-RPE capsid generated through in vivo-directed evolution efficiently targets RPE cells following IVT injection in mice and rhesus monkeys. (A) Schematic representation of in vivo-directed evolution used to generate AAV-RPE. (B) Three weeks after IVT injection with AAV-RPE-GFP (6 × 10^9^ vg/eye, *n* = 6 eyes) or AAV2.7m8-GFP (6 × 10^9^ vg/eye, *n* = 6 eyes); retinal sections from adult C57BL/6J mice showed that AAV-RPE-GFP was mainly expressed in RPE, while AAV2.7m8-GFP was mainly expressed in GCL. (C) Nine weeks after IVT injection with AAV-RPE-GFP (1.945 × 10^11^ vg/eye, *n* = 2 eyes; 3.89 × 10^11^ vg/eye, *n* = 2 eyes); the retinal sections of rhesus monkeys showed that GFP was mainly expressed in RPE and RCL. Retinal sections were examined by fluorescence microscopy. White arrows represent GFP expression. Scale bars, 100 μm. AAV, adeno-associated virus; DAPI, 4′,6-diamidino-2-phenylindole; GCL, ganglion cell layer; GFP, green fluorescent protein; IPL, inner plexiform layer; INL, inner nuclear layer; ITR, inverted terminal repeat; IVT, intravitreal; OPL, outer plexiform layer; ONL, outer nuclear layer; PCR, polymerase chain reaction; RCL, rod and cone layer; RGC, retinal ganglion cell; RPE, retinal pigment epithelium layer.

To generate an AAV capsid with high transduction efficiency for RPE cells following IVT injection, molecular AAV evolution was performed using DNA family shuffling of viral capsid (cap) genes. Cap genes from AAV serotypes 1 to 13 (AAV1 to AAV13) were randomly fragmented and reassembled into a full-length cap gene library using polymerase chain reaction (PCR). The resulting viral library was produced, IVT administered in mice, and subjected to iterative rounds of selection. After harvesting tissues, cap gene variants were collected, reassembled into new libraries, and reinjected. Following 3 to 4 cycles of selection, novel AAV capsids were identified and screened for RPE-specific tropism by assessing fluorescence in retinal sections after IVT injection of each AAV variant carrying the green fluorescent protein (GFP) transgene (Fig. 1A). Preliminary screening identified an AAV capsid with high transduction efficiency in RPE cells, designated as AAV-RPE. This capsid included 5 fragments derived sequentially from AAV1, AAV7, AAV2, AAV1, and AAV6, as well as an S430I mutation at the fourth segment. To characterize its transduction properties, AAV-RPE-GFP was compared to AAV2.7m8, a commonly used control, in C57BL/6J mice. Results showed that AAV2.7m8-GFP predominantly mediated GFP expression in ganglion cells, whereas the IVT-injected AAV-RPE-GFP mainly induced robust GFP expression in RPE cells (Fig. 1B). To validate the transduction role of AAV-RPE-GFP in NHPs, experiments were conducted in rhesus macaques. Similarly, IVT injection of AAV-RPE-GFP resulted in highly efficient GFP expression localized to RPE cells (Fig. 1C). These findings indicate that AAV-RPE is a novel and effective AAV capsid that targets RPE cells with high efficiency following IVT administration.

### Therapeutic effects of XMVA09 in a laser-induced CNV mouse model

**Fig. 2.**
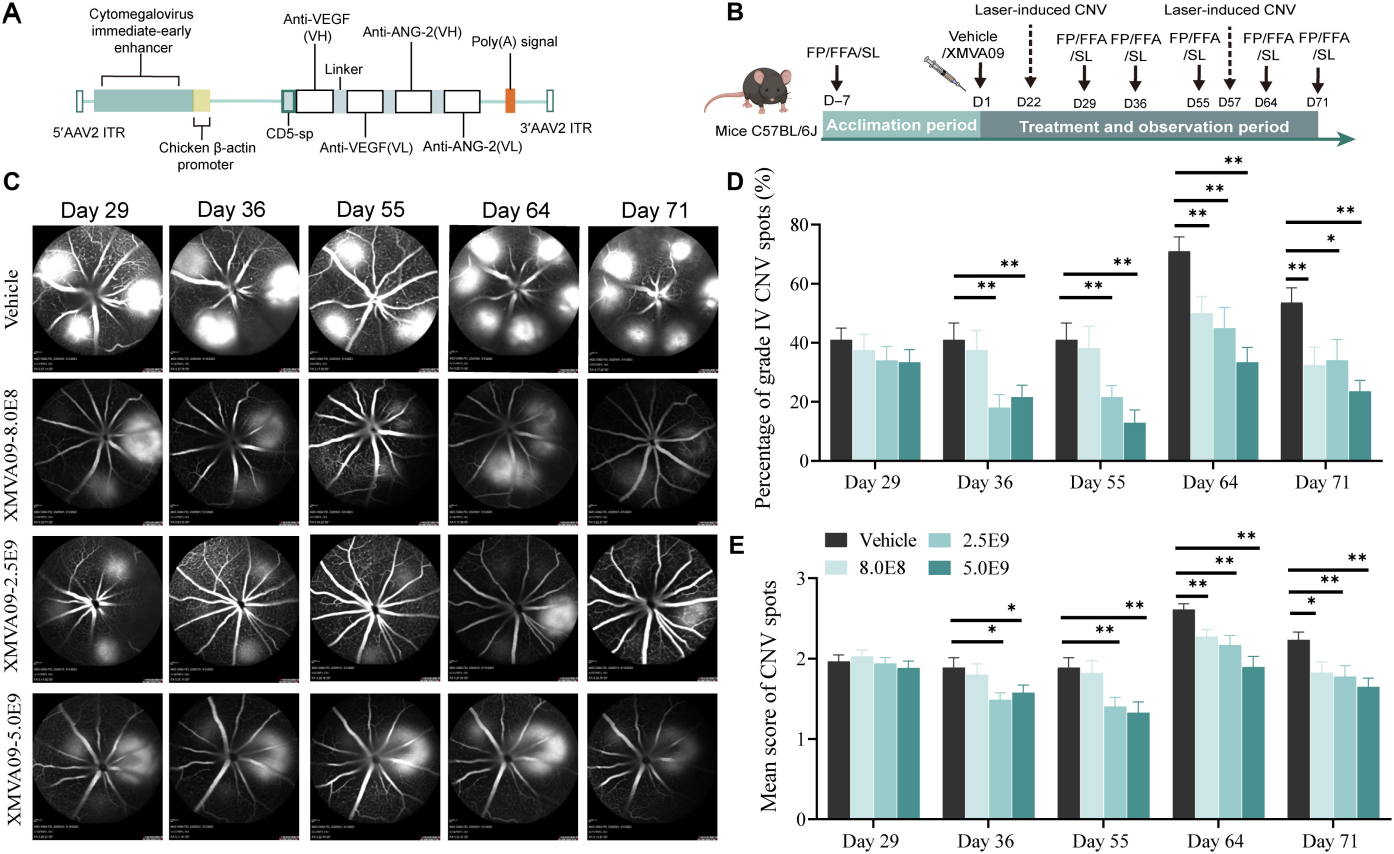
Efficacy of XMVA09 on laser-induced CNV lesions in mice. (A) Schematic of the XMVA09 genome. (B) Schematic of the study design. Adult C57BL/6J mice were randomized into vehicle and XMVA09 groups (8.0 × 10^8^, 2.5 × 10^9^, and 5.0 × 10^9^ vg/eye) and administered a single IVT injection in all eyes. Eyes were examined by SL, FP, and FFA before injection. On days 22 and 57, laser photocoagulation was performed in the bilateral fundus to induce CNV. SL, FP, and FFA examinations were performed on days (−7), 29, 36, 55, 64, and 71, and efficacy was evaluated based on the percentage of CNV grade IV lesions and mean score of CNV spots. (C) Representative FFA images of CNV spots in vehicle and XMVA09 groups. Fluorescence CNV spot grading definitions: grade I: no fluorescence leakage; grade II: mild fluorescence leakage, leakage area is 1% to 50% of the laser spot area; grade III: moderate fluorescence leakage, leakage area is 50% to 100% of the laser spot area; and grade IV: severe fluorescence leakage, leakage area is greater than laser spot size. (D) Changes in the percentage of grade IV CNV spots (from day 29 to day 71: vehicle: 40.97% ± 4.01%, 40.97% ± 5.67%, 40.97% ± 5.67%, 71.02% ± 4.83%, 53.62% ± 5.02%; XMVA09-8.0E8: 37.50% ± 5.33%, 37.50% ± 6.75%, 38.19% ± 7.34%, 50.00% ± 5.59%**, 32.45% ± 6.04%**; XMVA09-2.5E9: 34.03% ± 4.76%, 18.05% ± 4.36%**, 21.53% ± 3.95%**, 44.93% ± 7.06%**, 34.06% ± 6.99%*; XMVA09-5.0E9: 33.33% ± 4.37%, 21.53% ± 4.07%**, 12.88% ± 4.38%**, 33.33% ± 5.10%**, 23.53% ± 3.80%**). Spot ratio (%) = total number of spots of corresponding grade ÷ total number of spots of 4 types × 100%. (E) Changes in the mean score of CNV spots after injection (from day 29 to day 71: vehicle: 1.97 ± 0.08, 1.89 ± 0.12, 1.89 ± 0.12, 2.61 ± 0.07, 2.23 ± 0.10; XMVA09-8.0E8: 2.03 ± 0.08, 1.80 ± 0.13, 1.82 ± 0.15, 2.27 ± 0.09**, 1.83 ± 0.13*; XMVA09-2.5E9: 1.94 ± 0.08, 1.49 ± 0.09*, 1.40 ± 0.12**, 2.17 ± 0.12**, 1.78 ± 0.14**; XMVA09-5.0E9: 1.88 ± 0.09, 1.58 ± 0.09*, 1.33 ± 0.14**, 1.90 ± 0.13**, 1.65 ± 0.11**). Mean score of CNV spots = [(number of spots at grade I × 0) + (number of spots at grade II × 1) + (number of spots at grade III × 2) + (number of spots at grade IV × 3)] ÷ total number of spots. From day 29 to day 55, *n* = 24 eyes per group. On day 64, vehicle group (*n* = 23 eyes), XMVA09 groups (8.0 × 10^8^ vg/eye, *n* = 22 eyes; 2.5 × 10^9^ vg/eye, *n* = 23 eyes; 5.0 × 10^9^ vg/eye, *n* = 19 eyes). On day 71, vehicle group (*n* = 23 eyes), XMVA09 groups (8.0 × 10^8^ vg/eye, *n* = 19 eyes; 2.5 × 10^9^ vg/eye, *n* = 23 eyes; 5.0 × 10^9^ vg/eye, *n* = 18 eyes). Data are presented as mean ± standard error of the mean (SEM), with SEM shown as error bars. Compared with the vehicle group, *P < 0.05 and **P < 0.01. CNV, choroidal neovascularization; SL, slit lamp; FP, fundus photography; FFA, fundus fluorescein angiography; VEGF, vascular endothelial growth factor; ANG-2, angiopoietin-2.

The therapeutic effects of XMVA09, consisting of an AAV-RPE capsid and a transgene payload encoding a bispecific antibody against both VEGF and ANG-2 (Fig. 2A), were investigated in a laser-induced CNV mouse model. Different doses of XMVA09 (8.0 × 10^8^, 2.5 × 10^9^, and 5.0 × 10^9^ vg/eye) were administered via a single IVT injection on day 1 (Fig. 2B). Binocular fundus laser photocoagulation was performed on days 22 and 57, and ophthalmic examinations were performed on days 29, 36, 55, 64, and 71. Progression of CNV was assessed using fundus fluorescein angiography (FFA) to measure fluorescence leakage and classify the percentage of grade IV lesions based on the established grading criteria described in Table S1. The mean leakage scores and the percentage of leakage spots were used as the final effective endpoints for evaluating therapeutic efficacy (Fig. 2C to E). Results demonstrated that a single IVT injection of XMVA09 significantly reduced fluorescence leakage and exhibited a dose-dependent inhibition of laser-induced CNV in mice.

We also assessed the efficacy of XMVA09 (AAV-RPE-VEGF-Ang2; VEGF-Ang2 represents our specific dual-target antibody), AAV-RPE-aflibercept, AAV2.7m8-aflibercept, and AAV2.7m8 driving our specific dual target antibody VEGF-Ang2 through IVT injection in CNV mouse models. The mice were divided into 5 groups, the vehicle group, AAV-RPE-VEGF-Ang2 group, AAV-RPE-aflibercept group, AAV2.7m8-VEGF-Ang2 group and AAV2.7m8-aflibercept group. On day 1, the test samples and vehicle were delivered through IVT injection at a dosage of 5.0 × 10^9^ vg/eye. On day 22, CNV laser modeling was performed. On day 36, FFA and fundus photography (FP) inspection was performed. Our results showed that both AAV-injected groups could significantly inhibit laser-induced CNV and reduce fluorescence leakage (Fig. S1C and D). There was no significant difference between AAV-RPE-VEGF-Ang2 and AAV-RPE-Aflibercept or AAV2.7m8-VEGF-Ang2 in the percentage of grade IV CNV spots and the mean score of CNV spots, and significant decreases in the percentage of grade IV CNV spots and the mean score of CNV spots of AAV-RPE-VEGF-Ang2, AAV-RPE-Aflibercept, and AAV2.7m8-VEGF-Ang2 compared to those of AAV2.7m8-Aflibercept were observed (Fig. S1C and D). These results indicated the advantage of AAV-RPE and XMVA09 in CNV inhibition.

### Therapeutic effects of XMVA09 in a laser-induced CNV NHP model

**Fig. 3.**
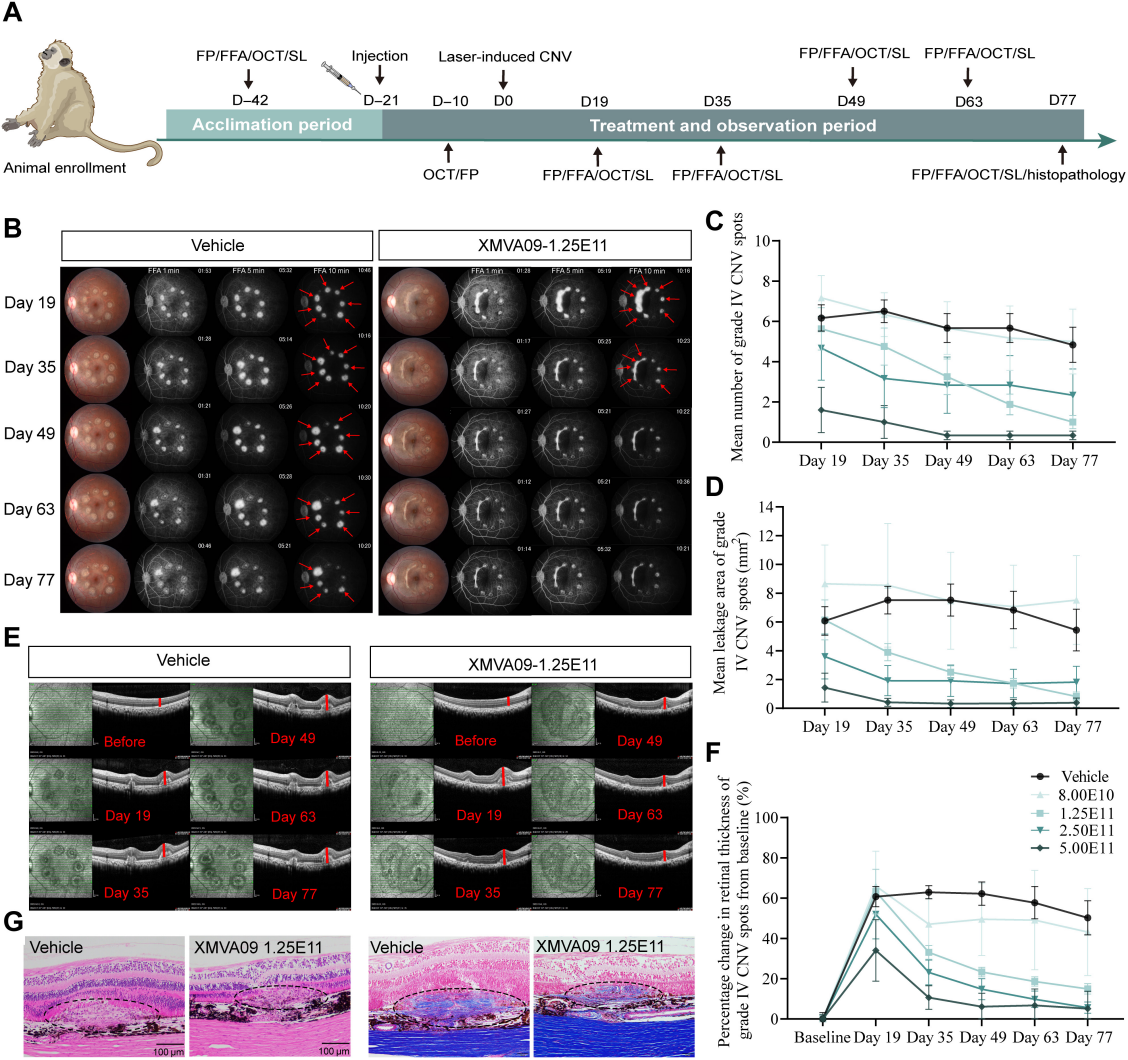
Efficacy of XMVA09 on laser-induced CNV lesions in rhesus monkeys. (A) Schematic of the study design. Rhesus monkeys were randomized into vehicle and XMVA09 groups (8.00 × 10^10^ vg/eye, *n* = 6 eyes; 1.25 × 10^11^ vg/eye, *n* = 8 eyes; 2.50 × 10^11^ vg/eye, *n* = 6 eyes; and 5.00 × 10^11^ vg/eye, *n* = 6 eyes) and administered a single IVT injection in all eyes. (B) Representative FFA images showing variation in the number of grade IV spots in vehicle and XMVA09 groups. Red arrows represent grade IV lesions (CNV spot grading definitions: grade I: no hyperfluorescence; grade II: hyperfluorescence without leakage; grade III: early hyperfluorescence or mid transit and late leakage; and grade IV: transit bright hyperfluorescence with leakage beyond borders of burned area). (C) Changes in the number of grade IV CNV spots. (D) Changes in the leakage area of grade IV CNV spots (from days 19 to 77: vehicle: 6.17 ± 0.65, 6.50 ± 0.56, 5.67 ± 0.71, 5.67 ± 0.71, 4.83 ± 0.87; 8.00 × 10^10^: 7.17 ± 1.11, 6.33 ± 1.09, 5.67 ± 1.31, 5.17 ± 1.60, 5.00 ± 1.61; 1.25 × 10^11^: 5.63 ± 1.05, 4.75 ± 0.92, 3.25 ± 0.88, 1.88 ± 0.52**^##^, 1.00 ± 0.33**^##^; 2.50 × 10^11^: 4.67 ± 1.59, 3.17 ± 1.45, 2.83 ± 1.40, 2.83 ± 1.47, 2.33 ± 1.31; 5.00 × 10^11^: 1.60 ± 1.12**, 1.00 ± 0.82**, 0.33 ± 0.21**, 0.33 ± 0.21**, 0.33 ± 0.21**). (E) Representative OCT images at baseline, as well as on days 19, 35, 49, 63, and 77 post-laser-induced CNV in vehicle and XMVA09 groups (the solid red line represents the retinal thickness of grade IV spots). (F) Percentage change from baseline in grade IV CNV spot retinal thickness in vehicle and XMVA09 groups (from baseline to day 77: vehicle: 60.72% ± 5.04%, 62.90% ± 3.26%, 62.22% ± 5.81%, 57.70% ± 8.01%, 50.20% ± 8.46%; 8.00 × 10^10^: 65.99% ± 17.31%, 47.01% ± 12.89%, 49.50% ± 17.98%, 49.11% ± 24.69%, 43.16% ± 21.57%; 1.25 × 10^11^: 63.29% ± 11.06%, 33.08% ± 3.40%**^#^, 23.29% ± 2.38%**^##^, 18.62% ± 2.30%**^##^, 14.95% ± 2.60%**^##^; 2.50 × 10^11^: 51.97% ± 12.23%, 23.09% ± 6.15%**, 14.76% ± 5.21%**^#^, 9.80% ± 4.06%**^##^, 5.65% ± 2.79%**^##^; 5.00 × 10^11^: 34.03% ± 15.28%, 10.60% ± 5.86%**, 6.14% ± 6.59%**, 6.77% ± 8.12%**, 5.09% ± 8.64%**). (G) Areas of hyperplasia of subretinal/choroidal fibrous tissue and focal retinal degeneration (black circles) were identified by hematoxylin and eosin (H&E) and Masson staining on day 77 after laser-induced CNV. CNV lesions (black dashed lines) were also identified based on H&E and Masson staining on day 77 after laser-induced CNV. H&E, scale bars, 100 μm. Masson, scale bars, 50 μm. Data are presented as mean ± SEM, with SEM shown as error bars. Compared with the vehicle group, *P < 0.05 and **P < 0.01. Compared with day 19, ^#^*P* < 0.05 and ^##^*P* < 0.01. OCT, optical coherence tomography.

The therapeutic potential of XMVA09 was further evaluated in rhesus macaques with laser-induced CNV. IVT injections of XMVA09 at various doses (8 × 10^10^, 1.25 × 10^11^, 2.5 × 10^11^, and 5 × 10^11^ vg/eye) were administered 21 d prior to CNV induction via laser photocoagulation. Ophthalmic evaluations, including FP, FFA, optical coherence tomography (OCT), and slit-lamp examinations, were performed before and after laser induction. CNV leakage grading based on FFA was conducted according to the criteria detailed in Table S2. Histological assessments using hematoxylin and eosin (H&E) and Masson’s trichrome staining were performed on CNV lesions at the end of the study (Fig. 3A).

Results showed that XMVA09 treatment (1.25 × 10^11^ vg/eye) significantly reduced the number of grade IV CNV lesions on days 63 and 77 compared to that of the vehicle-treated group (Fig. 3B and C and Table S3). Higher XMVA09 doses (5 × 10^11^ vg/eye) showed earlier therapeutic effects, with a reduction in grade IV CNV lesions by day 19 post-laser induction (Fig. 3C and Table S3). Additionally, the leakage area of grade IV CNV lesions was significantly reduced by XMVA09 treatment (1.25 × 10^11^ and 2.5 × 10^11^ vg/eye) from day 35 onward (Fig. 3D and Table S4). The highest dose (5 × 10^11^ vg/eye) exhibited a more pronounced reduction in leakage area, evident as early as day 19. Retinal thickness at grade IV CNV sites was also markedly decreased in XMVA09-treated eyes (1.25 × 10^11^, 2.5 × 10^11^, and 5 × 10^11^ vg/eye), starting from day 35 post-laser induction (Fig. 3F and Table S5). Histological analyses revealed that XMVA09 treatment alleviated CNV-induced hyperplasia of subretinal/choroidal fibrous tissue and focal retinal degeneration, as evidenced by H&E and Masson’s trichrome staining (Fig. 3G). Collectively, these findings indicate that a single IVT injection of XMVA09 effectively inhibits laser-induced CNV progression in rhesus monkeys, highlighting its potential as a therapeutic agent for neovascular ocular conditions.

### IVT injection of XMVA09 does not induce retinal toxicity in NHPs

The safety profile of XMVA09 was thoroughly evaluated in an independent study conducted in healthy cynomolgus monkeys. Animals were randomly assigned to 3 groups: the vehicle control group, XMVA09 low-dose group (1 × 10^11^ vg/eye), and XMVA09 high-dose group (2.5 × 10^11^ vg/eye). Clinical observations and ophthalmological examinations were performed for 89 d following IVT injection (Tables S6 and S7). Minimal to mild conjunctival congestion, swelling, and anterior chamber cells were observed in all groups on day 3 postinjection, which recovered by day 8 (Tables S6 and S7). Pigment-like particles of varying sizes were detected in the anterior vitreous body across all 3 groups, with the incidence and severity of these exudates remaining low and dose independent. These inflammatory responses, common with IVT injections, were considered mild and clinically insignificant.

**Fig. 4.**
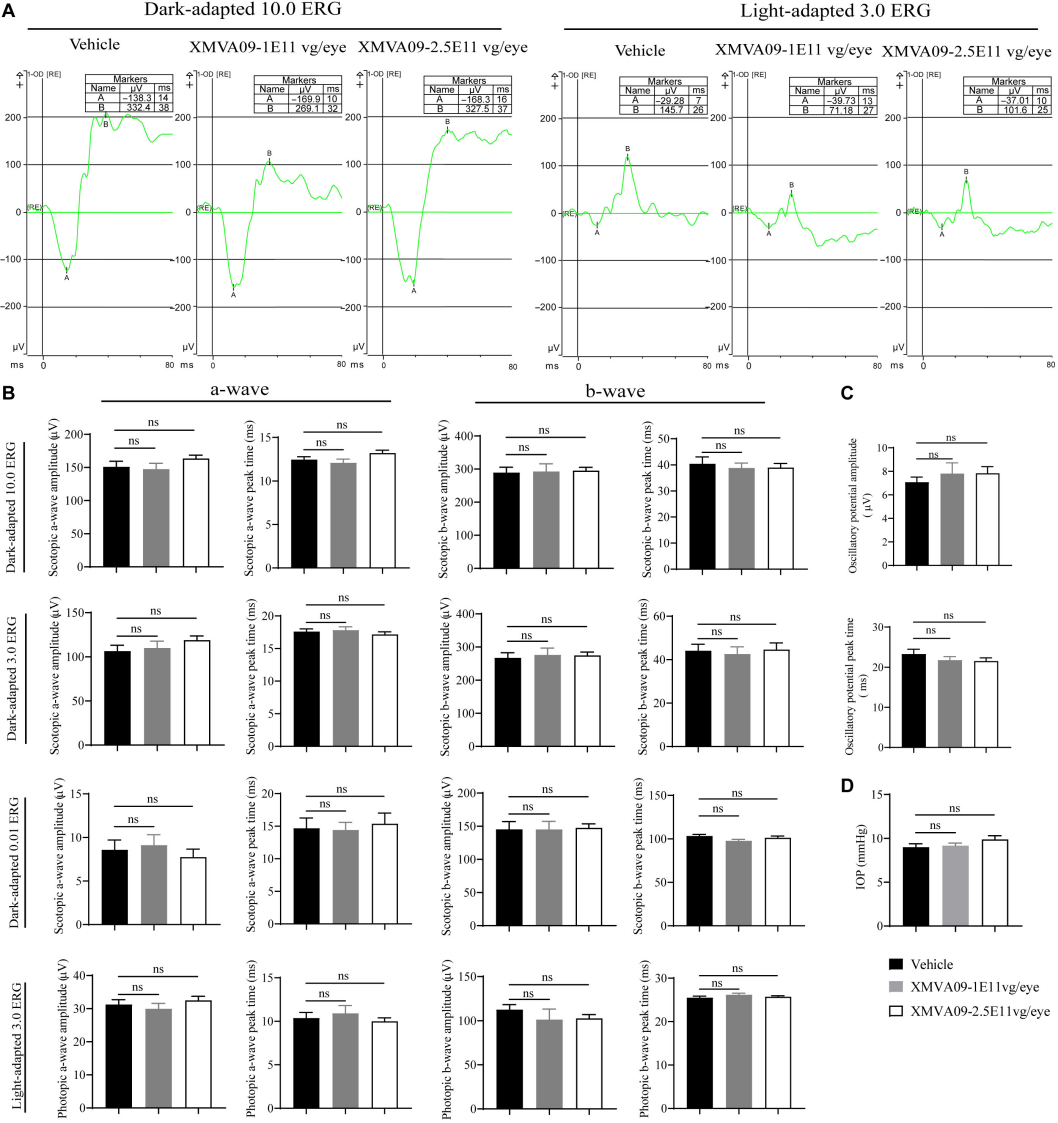
XMVA09 exerts no apparent retinal functional toxicity in cynomolgus monkeys. (A) Representative images of scotopic ERG (dark-adapted 10.0 ERG) and photopic ERG (light-adapted 3.0 ERG) in eyes treated with the vehicle and XMVA09. Vehicle group (*n* = 22 eyes) and XMVA09 groups (1 × 10^11^ vg/eye, *n* = 12 eyes; 2.5 × 10^11^ vg/eye, *n* = 24 eyes). Retinal function was assessed by ERG and IOP examinations on day 89 post-IVT injection. (B to D) Compared to the vehicle group, no apparent XMVA09-related abnormalities were observed in any eye. ERG, electroretinography; IOP, intraocular pressure. Statistical analysis was performed using *t* tests. Data are presented as mean ± SEM, with SEM shown as error bars. ns, no significance.

**Fig. 5.**
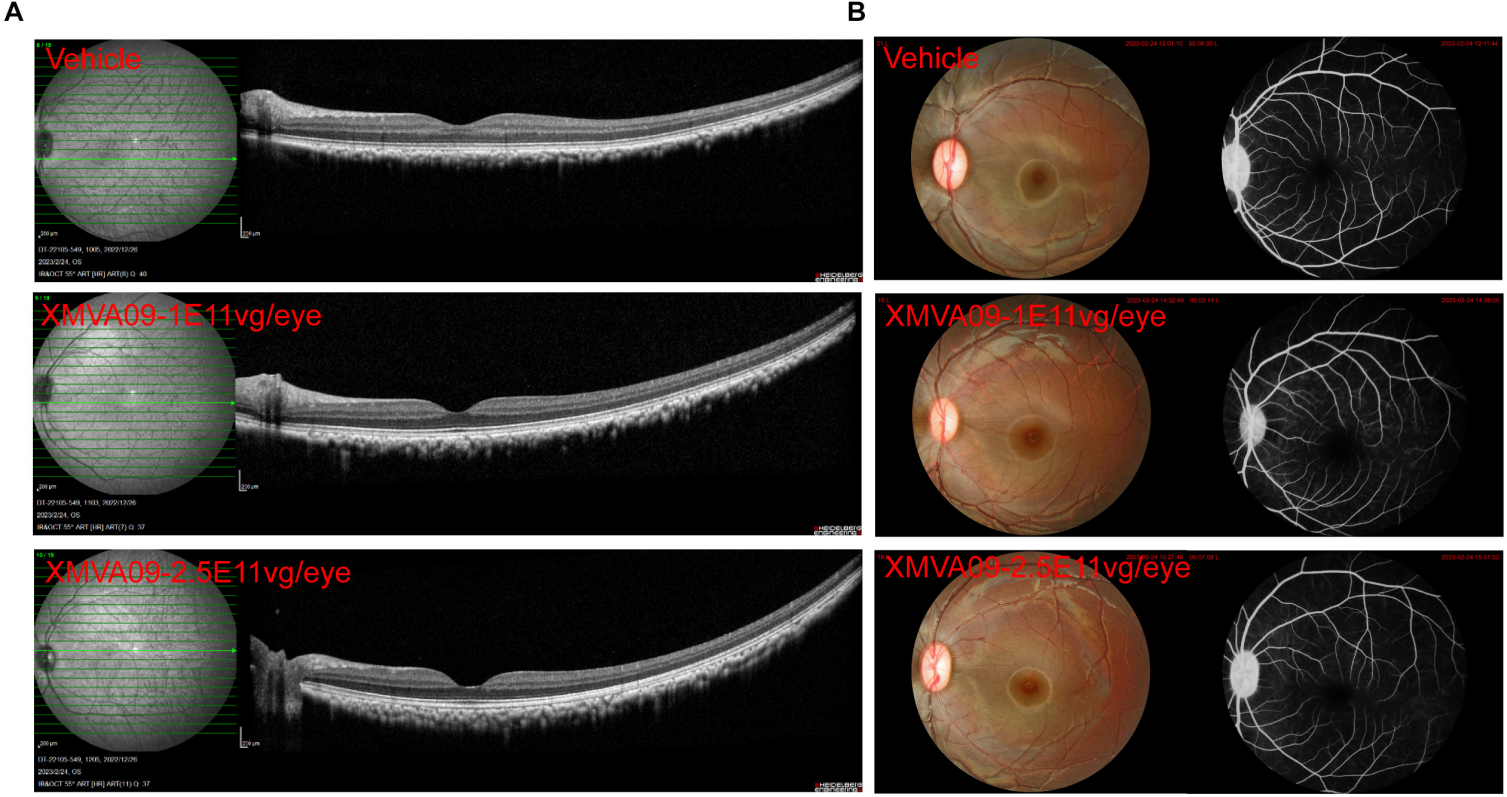
XMVA09 exerts no apparent abnormalities based on OCT and FFA in cynomolgus monkeys. (A) Representative images of OCT showing no marked differences in retinal morphology in XMVA09 groups compared to the vehicle group. Vehicle group (*n* = 22 eyes) and XMVA09 groups (1 × 10^11^ vg/eye, *n* = 12 eyes; 2.5 × 10^11^ vg/eye, *n* = 24 eyes). Retinal morphology was analyzed by OCT and FFA on day 89 post-IVT injection. (B) Representative images of FFA examination revealed no obvious changes in retinal vessels and optic disk between XMVA09 and vehicle groups.

To assess potential retinal toxicity, electroretinography (ERG) was performed to evaluate retinal function following XMVA09 treatment. Results showed that XMVA09 treatment did not cause any abnormalities in a-wave amplitudes and peak times or b-wave amplitudes and peak times (Fig. 4A to C and Table S8). Intraocular pressure (IOP) measurements further demonstrated no adverse effects of XMVA09 treatment on ocular health in cynomolgus monkeys (Fig. 4D). Retinal toxicity was also examined using OCT (Table S9), FP (Table S10), and FFA (Table S11). Results demonstrated that XMVA09 treatment did not alter retinal thickness in cynomolgus monkeys (Fig. 5A and B). Dot- and sheetlike hyperreflective signals were occasionally observed in the vitreous bodies by OCT in 2 eyes from the vehicle group, 1 eye from the low-dose group, and 1 eye from the high-dose group. These signals, along with the pigment-like particles identified during ophthalmic examinations, were attributed to the inflammatory responses caused by the injection procedure rather than XMVA09 itself. Overall, these findings indicate that XMVA09 is well tolerated following IVT administration, with no evidence of retinal toxicity or adverse effects on ocular function, even at higher doses.

### XMVA09 biodistribution in ocular and other tissues following IVT injection in NHPs

The biodistribution of the target gene and vector shedding were evaluated in cynomolgus monkeys after IVT injection of XMVA09 (2.5 × 10^11^ vg/eye) in both eyes. Analysis of ocular tissues revealed the presence of the target gene in the cornea, lens, iris–ciliary complex, retina, choroid, sclera, and optic nerve (Fig. S2A). In blood samples, no target gene was detected prior to dosing, but its presence was confirmed postinjection, with a clear elimination trend over time (Fig. S2B). Peripheral tissues and organs exhibited widespread target gene expression on days 31, 94, and 185 postinjection, with significant clearance observed over time (Fig. S2C). The target gene was also detected in oropharyngeal and nasopharyngeal swabs and urine, feces, and lacrimal samples from treated animals (Fig. S3).

Immunogenicity analysis demonstrated that a single IVT injection of XMVA09 induced the production of positive antibodies against AAV in the serum and vitreous humor in some dosed cynomolgus monkeys (Tables S12 and S13). Anti-AAV neutralizing antibodies were also observed in these compartments (Tables S14 and S15). Notably, no antibodies against the target protein were detected in either serum or vitreous humor throughout the study (Tables S16 and S17). These results suggested that XMVA09 is of low immunogenicity.

### Safety and tolerability of XMVA09 following IVT injection in nAMD patients

An investigator-initiated trial (IIT) was conducted to evaluate the safety and tolerability of XMVA09 treatment in patients with nAMD. The study enrolled 6 patients, divided into 2 groups (Table S18). The first group (*n* = 3) received a single IVT injection of XMVA09 at a dose of 8 × 10^10^ vg/eye, while the second group (*n* = 3) was administered a higher dose of XMVA09 (2.5 × 10^11^ vg/eye). Notably, unlike other clinical trials for nAMD gene therapies, participants in this study did not receive anti-VEGF drugs during the 4 weeks preceding XMVA09 administration.

Results indicated that XMVA09 was well tolerated, with no dose-limiting or drug-related adverse events reported (Table S19). Mild adverse events were observed in 3 patients, including dry eyes in both treated and fellow eyes in 2 patients and hyperbilirubinemia in 1 patient. All adverse events resolved without intervention. IOP elevations, a common side effect of IVT injections, were transient and nonsignificant in this study. The average pre-operation IOP was 15.92 mmHg, increasing by 2.3 mmHg (ranging from −4 to 8 mmHg) within 30 min postinjection. IOP levels returned to the normal range without requiring pressure-lowering medications. Overall, no marked XMVA09-related toxicity was observed, and the treatment was well tolerated across all patients. These findings suggest that IVT administration of XMVA09 exhibited a strong safety and tolerability profile in nAMD patients, supporting its potential as a therapeutic option for neovascular ocular diseases.

### Promising therapeutic efficacy of XMVA09 in nAMD patients without recent anti-VEGF treatment

**Fig. 6.**
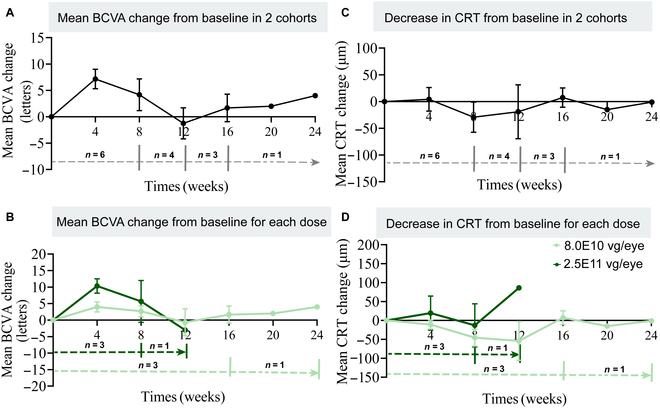
Functional and morphological changes in retina after treatment with XMVA09 in nAMD patients. (A) Mean BCVA change from baseline to week 24 postinjection (4 weeks: 7.17 ± 1.85, *n* = 6; 8 weeks: 4.17 ± 3.00, *n* = 6; 12 weeks: −1.25 ± 2.96, *n* = 4; 16 weeks: 1.67 ± 2.60, *n* = 3; 20 weeks: 2.00, *n* = 1; 24 weeks: 4.00, *n* = 1). (B) Mean BCVA change from baseline to week 24 for patients receiving each dose of XMVA09 (8.0 × 10^10^ vg/eye: 4 weeks: 4.00 ± 1.53, *n* = 3; 8 weeks: 2.67 ± 1.67, *n* = 3; 12 weeks: −0.67 ± 4.10, *n* = 3; 16 weeks: 1.67 ± 2.60, *n* = 3; 20 weeks: 2.00, *n* = 1; 24 weeks: 4.00, *n* = 1; 2.5 × 10^11^ vg/eye: 4 weeks: 10.33 ± 2.19, *n* = 3; 8 weeks: 5.67 ± 6.33, *n* = 3; 12 weeks: −3.00, *n* = 1). (C) Decrease in CRT from baseline to week 24 postinjection, as measured by macular OCT scans (4 weeks: 4.17 ± 21.97, *n* = 6; 8 weeks: −29.33 ± 28.29, *n* = 6; 12 weeks: −19.00 ± 50.51, *n* = 4; 16 weeks: 7.33 ± 18.10, *n* = 3; 20 weeks: −15.00, *n* = 1; 24 weeks: −1.00, *n* = 1). (D) Decrease in CRT from baseline to week 24 for patients that received each dose of XMVA09 (8.00 × 10^10^ vg/eye: 4 weeks: −11.00 ± 11.93, *n* = 3; 8 weeks: −45.67 ± 22.33, *n* = 3; 12 weeks: −54.00 ± 51.51, *n* = 3; 16 weeks: 7.33 ± 18.10, *n* = 3; 20 weeks: −15.00, *n* = 1; 24 weeks: −1.00, *n* = 1; 2.50 × 10^11^ vg/eye: 4 weeks: 19.33 ± 45.17, *n* = 3; 8 weeks: −13.00 ± 56.89 *n* = 3; 12 weeks: 86.00, *n* = 1). nAMD, neovascular age-related macular degeneration; BCVA, best corrected visual acuity, CRT, central retinal thickness. Data are presented as mean ± SEM, with SEM shown as error bars.

The preliminary efficacy of XMVA09 gene therapy was evaluated in patients with nAMD who had not received anti-VEGF treatment in the 4 weeks prior to the study. Efficacy was assessed by monitoring changes in best corrected visual acuity (BCVA) and central retinal thickness (CRT) from baseline. BCVA was well maintained over 24 weeks following XMVA09 treatment (Fig. 6A and B and Table S20), and CRT decreased in patients treated with low-dose XMVA09 within the same period (Fig. 6C and D and Table S20). These findings indicate that XMVA09 treatment stabilizes retinal condition and visual acuity in nAMD patients.

### Case 1

A 69-year-old female patient with a 3-year history of vision loss in the right eye participated in the study. The patient was diagnosed with exudative age-related macular degeneration in both eyes. Her medical history included hypertension and 3 prior anti-VEGF treatments, with no prior ocular surgeries or family history. Baseline examination revealed normal eye movement, a clear optic disk color boundary of the fundus in both eyes, and flat retinas. The macular area of the right eye displayed grayish-white lesions, while the left eye exhibited hemorrhagic macular features. The right eye was designated as the study eye, with a baseline BCVA of 30 letters. After 24 weeks of treatment, BCVA improved to 34 letters, while CRT increased marginally by 1 μm compared to baseline (Fig. S4). Subretinal fluid levels remained stable throughout the study, with no marked differences observed after XMVA09 injection.

### Case 2

A 68-year-old female presented with a 1-year history of bilateral vision loss. The patient was diagnosed with exudative age-related macular degeneration in both eyes. She had previously received 2 anti-VEGF treatments, with a medical history of liver cysts and resection but no prior ocular surgeries or relevant family history. Examination revealed normal eye movement in both eyes. The optic disks were flat bilaterally, with the macula of the left eye showing signs of chronic degeneration. XMVA09 was administered to the left eye, with a baseline BCVA of 63 letters. Following 16 weeks of treatment, BCVA improved to 69 letters (Fig. S5). CRT increased slightly by 1 μm from baseline, and subretinal fluid levels remained stable compared to pre-treatment conditions.

### Case 3

A 69-year-old male presented with a 4-month history of vision loss in the right eye. A diagnosis of nAMD was established for the right eye. His medical history included 3 prior anti-VEGF treatments, with no history of familial disease. Examination revealed normal eye movement in both eyes. The right eye fundus exhibited clearly defined structures, with slight macular edema and a small amount of exudate, while the left eye appeared normal. XMVA09 was administered to the right eye. Baseline BCVA was 70 letters, which decreased to 67 letters after 16 weeks of treatment. CRT also decreased from 386 μm at baseline to 344 μm posttreatment (Fig. S6). Subretinal fluid showed notable improvement, with a reduction in effusion compared to baseline.

### Case 4

A 69-year-old male presented with blurred vision in the right eye persisting for 1 year. The patient was diagnosed with exudative age-related macular degeneration in the right eye and macular edema in the left eye. His medical history included 3 prior anti-VEGF treatments, with no history of systemic or ocular surgeries or familial diseases. Ocular examination revealed normal eye movement bilaterally. The right eye fundus showed a clear optic disk, macular exudation, and a flat retina, while the left eye exhibited a clear optic disk boundary, macular edema with exudation, and a flat retina. XMVA09 was administered to the right eye, with a baseline BCVA of 67 letters. After 12 weeks of treatment, BCVA decreased slightly to 64 letters. CRT improved obviously, decreasing from 321 μm at baseline to 235 μm (Fig. S7). Subretinal fluid levels showed no marked changes from baseline.

### Case 5

A 55-year-old male presented with vision loss in the right eye for 6 months. His medical history included 3 prior anti-VEGF treatments, with no history of systemic or ocular surgeries or familial diseases. Examination revealed normal eye movements bilaterally. The right eye fundus displayed flat retinal structures and grayish-yellow macular lesions, while the left retina appeared flat. A diagnosis of nAMD was established for the right eye. XMVA09 (2.5 × 10^11^ vg/eye, 100 μl) was administered to the right eye, with a baseline BCVA of 45 letters. Following 8 weeks of treatment, BCVA improved to 57 letters. CRT increased slightly from 136 to 146 μm after treatment (Fig. S8). Subretinal fluid levels remained stable, with no significant changes from baseline.

### Case 6

A 60-year-old male presented with vision loss in the left eye for 6 months. His medical history included hypertension and 4 prior anti-VEGF treatments, with no history of systemic or ocular surgeries. Examination showed normal eye movements bilaterally, with flat retinal structures in both eyes and pigmentation disturbances in the left macular area. A diagnosis of nAMD was established for the left eye. XMVA09 was administered to the left eye, with a baseline BCVA of 55 letters. After 8 weeks of treatment, BCVA improved to 67 letters. CRT showed a marked reduction from 565 μm at baseline to 481 μm (Fig. S9). Subretinal fluid levels remained unchanged from baseline.

## Discussion

The current standard of care for nAMD involves IVT injections of anti-VEGF agents, typically administered monthly to maintain therapeutic efficacy [20–23]. However, this frequent dosing regimen imposes burdens on patients and caregivers while increasing the risks of complications, such as elevated IOP and endophthalmitis [24]. Even single-dose treatments, such as subretinal administration of Luxturna, have demonstrated a high incidence of ocular adverse events in clinical trials [25]. Furthermore, in a phase I/II clinical trial of the gene therapy candidate RGX-314, 42 patients with severe wet AMD requiring frequent anti-VEGF injections were stratified into 5 dose groups (ranging from 3 × 10^9^ to 2.5 × 10^11^ vg/eye). Intraocular inflammatory adverse events were observed in 66.67% (4/6), 50% (3/6), 50% (3/6), 25% (3/12), and 25% (3/12) of patients across the respective cohorts [15]. Similarly, in the phase I trial of the gene therapy candidate ADVM-022, ocular inflammation emerged as the most frequent adverse event, especially in the high-dose group, where it predominantly affected the anterior segment. Notably, no instances of posterior inflammation, vasculitis, or endophthalmitis were reported [26]. In contrast, our IIT of XMVA09 reported no injection-related adverse events, underscoring its favorable safety profile.

The efficiency, safety, and tissue specificity of AAV vectors have become key areas of interest in ocular gene therapy. AAV-mediated gene expression is influenced by multiple factors, including serotype and delivery route. For example, subretinal injection enables AAV1, AAV2, AAV6, AAV8, and AAV9 to efficiently transduce the outer nuclear layer and RPE, while IVT injection enables AAV2, AAV6, and AAV8 to target the inner retina [27]. However, compared to subretinal injection, IVT delivery offers marked advantages, as it is simpler and safer and can be performed in an outpatient setting [28]. Recent advancements in AAV capsid engineering have led to the development of novel capsids tailored for IVT injection for the treatment of ocular neovascular diseases, such as AAV2.7m8 and 4D-R100 [29]. However, despite its ability to transduce all retinal cell types in mice, AAV2.7m8 shows limited transduction of RPE cells in primates, primarily due to the thicker inner limiting membrane in primate retinas [29,30]. Conversely, 4D-R100 can successfully deliver transgenes across all major retinal layers in NHPs but exhibited low specificity [19]. Low specificity determines higher doses of AAV, indicating a higher incidence of health problems such as ocular inflammation [31,32]. This study established a novel AAV-RPE capsid capable of selectively targeting RPE cells in both mice and NHPs via IVT injection. This capsid enabled precise transgene delivery to RPE cells, making it particularly suitable for treating diseases involving lesions in the outer retina. Compared to subretinal RGX-314, a dose of 6 × 10^10^ or a higher genomic copy number resulted in stabilization or improvement in BCVA and CRT [15], while an IVT dose of XMVA09 with an 8 × 10^10^ genomic copy number achieved similar results by selectively targeting RPE. Minimize the need for high doses and reduce the risk of adverse events.

Despite their advantages, AAV vectors, including capsid proteins and nucleic acids, can activate both innate and adaptive immune responses, leading to cytokine release from neutrophils, B cells, and T cells [33,34]. In the nonclinical study of XMVA09 injection, most of the tested animals could produce positive antibodies against AAV in the serum after administration, and most cynomolgus monkeys produced anti-AAV neutralizing antibodies in the serum. After administration of a small amount to cynomolgus monkeys, positive antibodies against AAV and anti-AAV neutralizing antibodies can be produced in the vitreous humor. However, after administration, the anti-target protein antibodies in the serum and vitreous humor of the tested animals were negative. Despite this potential for immunogenicity, no immunogenicity-related adverse events have been identified in the current IIT studies of XMVA09. To mitigate risks, we implemented preventive measures and rigorous monitoring protocols. Patients who had previously received topical or systemic gene or cell therapy products, participated in clinical trials of such therapies, or were currently receiving immunosuppressants or immunoboosting agents were excluded from this trial. All subjects underwent regular immunogenicity testing and blo od tests (including blood routine and biochemistry) during the trial, with increased examination frequency if immunogenicity-related events occurred. Long-term follow-up studies are also being conducted to monitor immunogenicity-related events. If an immunogenicity-related event occurs, appropriate medical treatment will be administered according to the subject’s condition in a timely manner.

An important challenge in nAMD treatment is the variability in patient responses to anti-VEGF antibody therapy [35–38]. A substantial proportion of nAMD patients—estimated at 25% to 35%—exhibit inadequate responses to existing anti-VEGF agents [20]. Preclinical studies have demonstrated that dual inhibition of VEGF-A and ANG-2 is more effective at reducing neovascular leakage compared to monotherapies targeting either molecule alone [39]. Clinical trials of bispecific antibodies targeting both VEGF-A and ANG-2 have further validated these findings, showing improved safety and tolerability profiles along with extended efficacy in nAMD patients [40,41]. ANG-2 has emerged as a promising therapeutic target, particularly for patients who exhibit resistance or poor response to existing anti-VEGF therapies. Results from the TENAYA and LUCERNE phase 3 trials evaluating dual Ang-2 and VEGF-A inhibition with IVT faricimab, administered at up to 16-week intervals, demonstrated vision benefits and anatomical outcomes comparable with those of VEGF pathway inhibition alone with aflibercept given at 8-week intervals [42]. Extended durability of the effect with faricimab was observed, likely driven by the vascular-stabilizing effects of dual Ang-2 and VEGF pathway inhibition. Faricimab was considered noninferior to aflibercept (ClinicalTrials.gov, TENAYA NCT03823287 and LUCERNE NCT03823300). In our preclinical study in CNV mouse models, our findings align with clinical observations that the CNV inhibitory activity of AAV-RPE-VEGF-Ang2 is noninferior to that of AAV-RPE-aflibercept as well as the clinically tested AAV vector AAV2.7m8-VEGF-Ang2. XMVA09 may be a novel and safer therapeutic option for nAMD, with dual VEGF-A and ANG-2 targeting providing broader, sustained benefits, including for patients resistant to anti-VEGF treatments.

Furthermore, anti-VEGF monotherapies such as ranibizumab and aflibercept reduce CNV but incompletely suppress subretinal fibrosis (SRF) progression. Clinical trials report SRF incidence in patients despite regular dosing, potentially due to persistent inflammation or alternative fibrotic pathways [43,44]. Bispecific antibodies such as faricimab targeting VEGF-A/Ang-2 have shown better SRF inhibition in preclinical CNV models compared to anti-VEGF alone, highlighting dual pathway inhibition as a strategy to alleviate fibrosis [45]. In a preclinical monkey model, XMVA09 showed the potential to inhibit SRF. This may be due to the bispecific targeting of XMVA09 at work, as available data suggest that VEGF-A is inhibited to control neovascularization, while Ang-2 is blocked to disrupt the fibrotic pathway [46]. Future studies will combine longitudinal OCT and histopathological analysis to directly assess SRF outcomes in the XMVA09 treatment cohort.

In summary, XMVA09 represents an innovative gene therapy utilizing a targeted AAV-RPE capsid to deliver a bispecific antibody against both VEGF-A and ANG-2. This dual-target gene therapy, administered via IVT injection, has demonstrated efficacy in maintaining vision and tolerability in nAMD patients. XMVA09 offers a safe, effective, and economically accessible treatment option, presenting a substantial advancement in the management of nAMD.

## Materials and Methods

### Drug design and generation

XMVA09 is an AAV gene therapy encoding bispecific antibodies targeting VEGF and ANG-2. The AAV capsid was designed to enable stable expression of VEGF-A/ANG-2 bispecific antibodies in the eye via IVT injection. XMVA09 was produced by PackGene Biotech Inc. (Guangzhou, China) in compliance with institutional quality standards.

### Construction of DNA family shuffling library and in vivo screening

DNA family shuffling was performed with the capsid sequences of 13 AAV serotypes (AAV1, AAV2, AAV3, AAV4, AAV5, AAV6, AAV7, AAV8, AAV9, AAV10, AAV11, AAV12, and AAV13) found in human and nonhuman primates as parents. A total of 4 μg of the parental capsid sequences were mixed in equal molar ratios for DNA family shuffling and treated with 0.04 U of DNase I at 25 °C for 30 s to randomly break the complete parent capsid sequences into fragments of different lengths. DNA fragments with a size of 100 to 500 bp were recovered and purified, and 500 ng of DNA fragments was subjected to primer-less PCR to extend to a complete chimeric capsid sequence. After sufficient chimeric capsid sequences were obtained by amplification, the chimeric capsid sequences were recombined to a library vector containing an AAV2 Rep gene and an inverted terminal repeat sequence, and the DNA family shuffling library plasmid was extracted. Viral packaging was performed, and then virus titer was determined by quantitative real-time polymerase chain reaction (qPCR) using AAV2 Rep gene-specific primers (WPRE-F: GTCAGGCAACGTGGCGTGGTGTG; WPRE-R: GGCGATGAGTTCCGCCGTGGC). Approximately 1 × 10^10^ to 1 × 10^11^ vg of library virus was injected into mice; after 7 to 10 d of injection, the mice were euthanized, and the tissues were collected. The total tissue DNA was extracted by TRIzol, and the capsid genes of AAVs were recovered from the total DNA using the PCR method to complete an in vivo screening of the AAV library. The fragments obtained by PCR were cloned into the library vector to obtain the next round of library plasmid, and the library plasmid was packaged into a virus again, so that the in vivo screening process could be repeated, and after 3 to 5 rounds of screening, the recovered capsid genes of AAVs were subjected to 3-generation sequencing. The sequence of the encoding gene obtained from the above sequencing result was synthesized by GENEWIZ, and a genomic plasmid containing GFP was constructed. Viral packaging was performed to obtain the novel AAV viral variants AAV-RPE-GFP.

### Packaging of AAV

The plasmid encoding the gene of interest and the adenovirus helper plasmid and AAV-RPE capsid plasmid were cotransformed into HEK293T cells in appropriate quantities. The culture medium was collected after transfection for 72 h, and both cells and medium were harvested after an additional 48 h. Cells were lysed with 110 mM citrate buffer (pH 4.2) to release the AAV particles. The virus-containing supernatant was neutralized by adding one-fifth volume of 2 M HEPES. Following centrifugation, 8% polyethylene glycol 8000 and 500 mM sodium chloride were added, and the mixture was incubated at 4 °C overnight to precipitate the virus. After centrifugation, the precipitate was resuspended in phosphate-buffered saline containing 2 mM Mg^2+^ and treated with 100 U/ml Benzonase for nucleic acid digestion at 37 °C for at least 1 h. The virus suspension was purified by ultracentrifugation with iodixanol density gradient solution (15%, 25%, 40%, and 60%) to obtain the AAV.

### Animals

Male wild-type C57BL/6J mice (5 to 7 weeks old) were obtained from Shanghai Jihui Laboratory Animal Feeding Co., Ltd. (China). The mice were housed in a climate-controlled animal facility under a 12-h light/dark cycle with free access to food and water.

Rhesus monkeys (3 to 6 years old) were provided by Ya’an Primed Biotech Co., Ltd. (China), and cynomolgus monkeys (3 to 5 years old) were obtained from Guangxi Hechideheng Biotechnology Co., Ltd. (China). The monkeys were housed in a temperature- (18 to 26 °C) and humidity-controlled (40% to 70%) facility under a 12-h light/dark cycle with free access to food and water. All animal welfare procedures and experiments complied with the relevant policies and guidelines of the testing facilities and adhered to the standards set by the Animal Care Committee of the government. Throughout the study, animals were clinically monitored, and any exhibiting severe symptoms of discomfort were appropriately treated. Animals that were dying were euthanized in accordance with ethical guidelines.

### IVT injection

Mice were anesthetized via intramuscular injection of Zoletil 50 (50 mg/kg, 50 mg/ml), and pupil dilation was achieved using 0.5% compound tropicamide eye drops. Prior to anesthesia, 1 to 2 drops of mydriatic solution were applied to each eye. Anesthetized mice were positioned laterally on the operating table, and the periorbital skin was disinfected with povidone-iodine cotton balls. The eyeball was fully exposed, and a puncture site was selected 1 to 2 mm posterior to the sclera limbus in the upper temporal or superior nasal region of the eye. A disposable sterile injection needle was used to puncture and establish a drug delivery channel. A micro-sampler needle (syringe, Hamilton needle, 7632-01) was inserted through the puncture channel into the vitreous cavity to deliver XMVA09 or the vehicle. Care was taken to avoid damaging the posterior lens capsule or retinal structures. After the injection, the needle was withdrawn slowly following a 10-s pause, and erythromycin eye ointment was applied to the eye. The mice were returned to their cages, and normal activity was monitored within 4 h postprocedure.

Before dosing, mydriatic eye drops were applied to the rhesus and cynomolgus monkeys. The rhesus monkeys were anesthetized with intramuscular ketamine (10 mg/kg) followed by intravenous propofol (0.5 to 1.0 ml/kg), while cynomolgus monkeys were anesthetized with intramuscular Zoletil 50 (5 mg/kg, 50 mg/ml). After anesthesia, the monkeys were placed on their backs. The palpebral margins, eyelashes, and surrounding skin and fur were disinfected with povidone-iodine solution, and eyeballs were fully exposed. The recommended injection site was located at 2 to 3 mm posterior to the temporal or supranasal corneoscleral margin. A sterile needle was inserted into the vitreous cavity to administer the injection while avoiding damage to the posterior lens capsule and retinal tissues. After the needle was withdrawn, a cotton swab was applied to the injection site for hemostasis.

### Laser-induced CNV

Laser-induced CNV lesions were created in mice on days 22 and 57 at 3 locations per eye. The laser parameters were as follows: laser wavelength 532 nm, spot size 50 μm, energy 200 mW, and time of exposure 0.1 s. After pupil dilation, carbomer eye drops were applied to improve visualization. A fundus laser was positioned in front of the eye to clearly observe the fundus, and photocoagulation was performed around the optic papilla at distance of about 1.5 to 2 PD from the optic disk.

Experimental CNV lesions in rhesus monkeys were induced using a laser photocoagulation system (Vitra 532 nm, Quantel Medical, France). The laser parameters followed standard protocols: laser wavelength 532 nm, spot size 50 μm, energy 650 to 700 mW, and time of exposure 0.1 s. Monkeys were anesthetized with intramuscular ketamine hydrochloride (20 mg/kg) and dexmedetomidine hydrochloride (0.03 mg/kg). Nine laser spots were applied around the macula in each eye, space approximately one disk diameter from the fovea. Care was taken to avoid injuring the fovea. The laser energy was adjusted based on the formation of subretinal blisters, which indicated successful puncture of Bruch’s membrane. If no blister formed, higher energy levels were applied for subsequent spots.

### General ophthalmology examination

The anterior segment of each animal was examined using a slit lamp, assessing structures including the eyelid, conjunctiva, cornea, anterior chamber, iris, sclera, pupil, pupillary light reflex, lens, and vitreous body. The fundus was evaluated using indirect ophthalmoscopy.

### Optical coherence tomography

The monkeys were immobilized under sedation, ensuring that their eyes remained open and heads stayed in position. OCT scans were performed using the Heidelberg Spectralis OCT Plus system by an experienced physician. When the macular fovea was focused by observation on the monitor screen, the fast macular scan procedure was applied to check the eyes of the monkeys. Retinal thickness by determining the distance between the inner limiting membrane and the base membrane was measured automatically by the built-in software of Heidelberg OCT. Locating the 2 membrane lines manually is necessary. The location with the maximal change of thickness at one of the CNV spots after laser induction was chosen for measurement. Heidelberg OCT has unique technical characteristics ensuring longitudinal measurement of retinal thickness at the same location.

### Fundus photography

FP was performed using a retinal camera (TRC-50DX, Topcon Healthcare, Japan). The working distance and focus were carefully adjusted before capturing images to ensure clarity.

### Fundus fluorescein angiography

For the mouse studies, fluorescein sodium (100 mg/ml, 0.02 ml) was rapidly injected into the tail vein of mice, and several clear images were captured for both eyes. Early-phase (1.5 min) and late-phase (>5 min) FFA images were compared to assess fluorescein leakage. Leakage spots were rated according to specified criteria (Table S1), and the ratio of leakage spots at each level and mean leakage score were calculated.

For rhesus monkey studies, 10% fluorescein sodium (Alcon Laboratories, USA) was injected intravenously at a dose of 0.075 ml/kg. Sequential photographs of the posterior poles were taken at specific time points, including early phase (1 min) and late phase (5 and 10 min), to monitor fluorescein leakage associated with CNV lesions. FFA images were graded for CNV lesions, and the number of grade IV CNV spots was determined (Table S2). Late-phase (10 min) FFA images were analyzed using the ImageJ software (v1.53e, National Institutes of Health, Wayne, Rasband, USA) to quantify the fluorescein leakage area of grade IV CNV spots.

### Electroretinography

Animals underwent dark-adapted ERG assessments, including dark-adapted 10.0 ERG and dark-adapted 3.0 ERG with oscillatory potentials, after adaptation in a dark room for at least 30 min. This was followed by light-adapted ERG (light-adapted 3.0 ERG) after at least 10 min of light adaptation. Full-field ERG data were collected using the Espion E3 System, exported as CSV files, and analyzed using Provantis v10.5.0 for statistical analysis and table generation.

### Intraocular pressure

IOP was measured using a tonometer. After general anesthesia induction, each monkey was secured in a seated position, and IOP was assessed by an experienced examiner.

### H&E staining

Under intravenous anesthesia of pentobarbital sodium (50 mg/kg), rhesus monkey eyeballs were rapidly enucleated and fixed in formalin-aceto-alcohol fixative for at least 24 h. The posterior pole containing visible laser spots was dissected, flattened, dehydrated, and embedded in paraffin. Laser spots used for retinal thickness measurements were selected for analysis. Paraffin blocks were sectioned into 5-μm slices across the designated laser spots and mounted on glass slides. The slides were dewaxed in xylene twice for 10 min each, followed by rehydration in graded alcohol (100%, 95%, 85%, and 75%) for 3 min each concentration, and finally in distilled water for 3 min. Sections were stained with hematoxylin solution for 18 min and rinsed under running tap water at room temperature for at least 5 min. Differentiation was performed using differentiation solution for 15 to 30 s, followed by washing with tap water at room temperature for 15 min. Slides were stained with eosin Y solution for 10 min, rinsed in running tap water at room temperature for at least 3 min, dehydrated in alcohol (95% and 100%), and cleared with 2 changes of xylene (10 min each). Permount was applied to the tissue, and coverslips were added. Slides were examined using a microscope.

### Masson’s trichrome staining

Paraffin sections were deparaffinized using distilled water. Slides were stained with Ponceau-Acid Fuchsin solution for 5 to 10 min, rinsed with distilled water, and differentiated in phosphomolybdic acid solution for 1 to 2 min or until collagen fibers were no longer red. Without rinsing, slides were stained with aniline blue solution for 1 to 2 min and then immersed in acetic acid working solution (1 part acetic acid solution and 2 parts distilled water) for 1 min. Slides were dehydrated in 95% alcohol and absolute alcohol, followed by clearing in xylene. A drop of Permount was applied to the tissue, and coverslips were added. Slides were examined using a microscope.

### Biodistribution and vector shedding

For cynomolgus monkey studies, samples from the XMVA09 (2.5 × 10^11^ vg/eye) group were collected to assess biodistribution and vector shedding. Blood samples were obtained from surviving animals pre-dose and on days 1 (2 h post-dose), 3, 6, and 30. Oropharyngeal and nasopharyngeal swabs and urine, feces, and lacrimal samples were collected pre-dose and on days 2, 3, 6, and 30. Tissues were collected on days 31, 94, and 185. Ocular tissues were collected from 3 animals on day 185. A validated qPCR method was used to detect the target gene in human cynomolgus monkey blood, tissue, and shedding samples. qPCR primer pairs (forward 5′-TGGCATGGTACCAGCAGAAG-3′, reverse 5′-ATACAGGAAAGAGGCGGAGTAGATC-3′) and a probe (5′-FAM-TGGCAAGGCCCCAAAGCTGC-BHQ-3′) were designed based on the XMVA09 target gene and synthesized by Zixi Biotechnology Co., Ltd. (China). After DNA extraction, qPCR was performed using AceQ Universal U+ Probe Master Mix V2 (Vazyme, China). Fluorescence signals from the probe were monitored to generate amplification curves. The threshold value was set to calculate the cycle threshold (CT) value for each reaction well, corresponding to the amplification cycle when the fluorescence signal reached the threshold. Data collected using QuantStudio qPCR v1.2 were exported in xls format for further analysis. Calibration curve fitting and sample concentration determination were performed in Microsoft Excel. A linear calibration curve (*Y* = *aX* + *b*) was constructed, where the *X* axis represents the logarithm (lg) of the XMVA09 target gene concentration (copies/5 μl) in calibration standards and the *Y* axis represents the CT values. The concentration of the target DNA in the samples was back-calculated using the CT values. Biodistribution was assessed by quantitatively determining the target gene content in cynomolgus monkey blood, tissue, and shedding samples.

### Immunogenicity

Samples from cynomolgus monkeys in vehicle and XMVA09 groups (1 × 10^11^ and 2.5 × 10^11^ vg/eye) were collected at specified time points to assess immunogenicity, including the detection of anti-AAV, anti-AAV neutralizing, and anti-target protein antibodies. Serum samples were collected pre-dose and on days 23, 50, 86, and 177, while vitreous humor samples were collected from both eyes on days 31, 94, and 185. The enzyme-linked immunosorbent assay was used for the detection of anti-AAV and anti-target protein antibodies in serum and vitreous humor. Detection of anti-AAV antibodies was performed using Empty Capsids (PackGene, China), Bio-AAV Empty Capsids-08 (TriApex, China), and streptavidin–horseradish peroxidase (Jackson ImmunoResearch, USA) with rabbit anti-AAV-M9 antibody (GenScript) used as a positive control. Detection of anti-target protein antibodies was performed using rabbit anti-XMVA09 protein polyclonal antibody (Biointron) and horseradish peroxidase–Protein G (GenScript, China), with XMVA09 protein (Biointron, China) as a positive control. A cell-based assay was used to detect anti-AAV neutralizing antibodies in serum and vitreous humor from samples testing positive for anti-AAV antibodies. The assay was performed using AAV carrying a luciferase reporter (PackGene, China), with anti-AAV empty capsid rabbit serum antiserum R14021#(3rd) (GenScript, China) as a positive control.

### Investigator-initiated trial

The clinical study enrolled 6 patients diagnosed with nAMD, who were treated and followed up at the First Affiliated Hospital of the University of Science and Technology of China. At each scheduled follow-up, patients underwent comprehensive eye examinations of both eyes, including FFA (Heidelberg Spectralis HRA) and OCT (RTVue XR 32075) to evaluate lesion areas.

### Ethics

The study complied with the Declaration of Helsinki and other relevant regulations of the World Medical Congress. Ethical approval was obtained from the institutional ethics committee before the study began. Prior to enrollment, investigators provided participants or their legal representatives with detailed explanations of the purpose, procedures, and potential risks of the study. Written informed consent was obtained, and participants were informed of their right to withdraw from the study at any time. Informed consent documents were retained as clinical study records. Participant privacy and data confidentiality were strictly maintained throughout the study.

### Participants

Participants included males and females aged 50 to 80 years (inclusive) with a confirmed diagnosis of nAMD and a history of IVT anti-VEGF therapy. Eligible study eyes were required to meet the following criteria: (a) active subfoveal or parafoveal CNV secondary to AMD, (b) BCVA ranging from 78 to 19 Early Treatment Diabetic Retinopathy Study letters (Snellen equivalent 20/32 to 20/400), (c) at least 2 anti-VEGF injections within 6 months prior to enrollment, (d) a CRT reduction of ≥50 μm in response to anti-VEGF therapy, (e) total lesion area (including hemorrhage, scarring, and neovascularization) ≤12 optic disk areas, and (f) absence of marked media opacity or miosis affecting fundus examination. Key exclusion criteria included the following: (a) the presence of retinal or choroidal diseases causing CNV other than AMD or diagnosed polypoidal choroidal vasculopathy deemed unsuitable for inclusion by the investigator; (b) active ocular infection; (c) uncontrolled glaucoma or defined as intraocular or severe glaucoma or previous glaucoma filtration surgery; (d) previous or current surgical or laser treatment involving the macula, deemed unsuitable for inclusion by the investigator; (e) history of vitreous hemorrhage within 3 months of screening, or intraocular surgery or laser treatment within 4 weeks of screening, or presence of intraocular implants or fillers; and (f) IVT/intrachoroidal injection of corticosteroids within 3 months of screening or long-acting IVT implant within 6 months of screening. Further details of the exclusion criteria are provided in the Supplementary Materials and Methods.

### Data processing and statistical analysis

Statistical analyses were performed using GraphPad Prism v8.0.2, Microsoft Excel, and SAS in Provantis v10.5.0. Data were initially assessed for normality and homogeneity of variance using the Shapiro–Wilk and Bartlett tests, respectively. For datasets with *P* ≥ 0.05, parametric tests were applied. For data exhibiting *P* < 0.05, Provantis v10.5.0 automatically transformed the data, followed by reapplication of the Shapiro–Wilk and Bartlett tests. If the transformed data yielded *P* ≥ 0.05, parametric tests were performed. For parametric analysis, one-way analysis of variance was performed. If no significant differences were detected (*P* ≥ 0.05), statistical analysis was terminated. For datasets exhibiting significant differences (*P* < 0.05), post hoc analysis was performed using the Dunnett test, with significant results marked on the mean (* or **) (*P* < 0.05). If the transformed data remained *P* < 0.05, this indicates that the data needed to be further analyzed by a nonparametric test. Data were analyzed by Kruskal–Wallis test. For result exhibiting no difference (*P* ≥ 0.05), the statistical analysis was terminated. For result exhibiting a difference (*P* < 0.05), post hoc analysis was performed using the Wilcoxon test, with significant results marked on the mean (* or **) (*P* < 0.05). An independent samples *t* test was also used to compare the group differences. For data exhibiting no difference (*P* ≥ 0.05), the statistical analysis was completed. For data exhibiting a difference (*P* < 0.05), the results were indicated on the mean (* or **).

## Data Availability

All data supporting the results of this study can be obtained from the corresponding authors upon reasonable request.
