# Supplementary material for: An Engineered Intravitreal Injection Retinal-Pigment-Epithelium-Tropic Adeno-Associated Virus Vector Expressing a Bispecific Antibody Binding VEGF-A and ANG-2 Rescues Neovascular Age-Related Macular Degeneration in Animal Models and Patients
Source: Research (Wash D C). 2025 May 29;8:0717. doi: 10.34133/research.0717 (PMC12120248; doi:10.34133/research.0717)
Supplement: Supplementary 1 — Figs. S1 to S9 Tables S1 to S20 Supplementary Materials and Methods [file research.0717.f1.docx]

**An engineered intravitreal injection RPE-tropic AAV vector expressing a bispecific antibody binding VEGF-A and ANG-2 rescues nAMD in animal models and patients**

**Supplementary Materials**

The supplementary materials are as follows:

**Fig. S1-S9**

**Table S1 to S20**

**IIT trial Materials and methods**


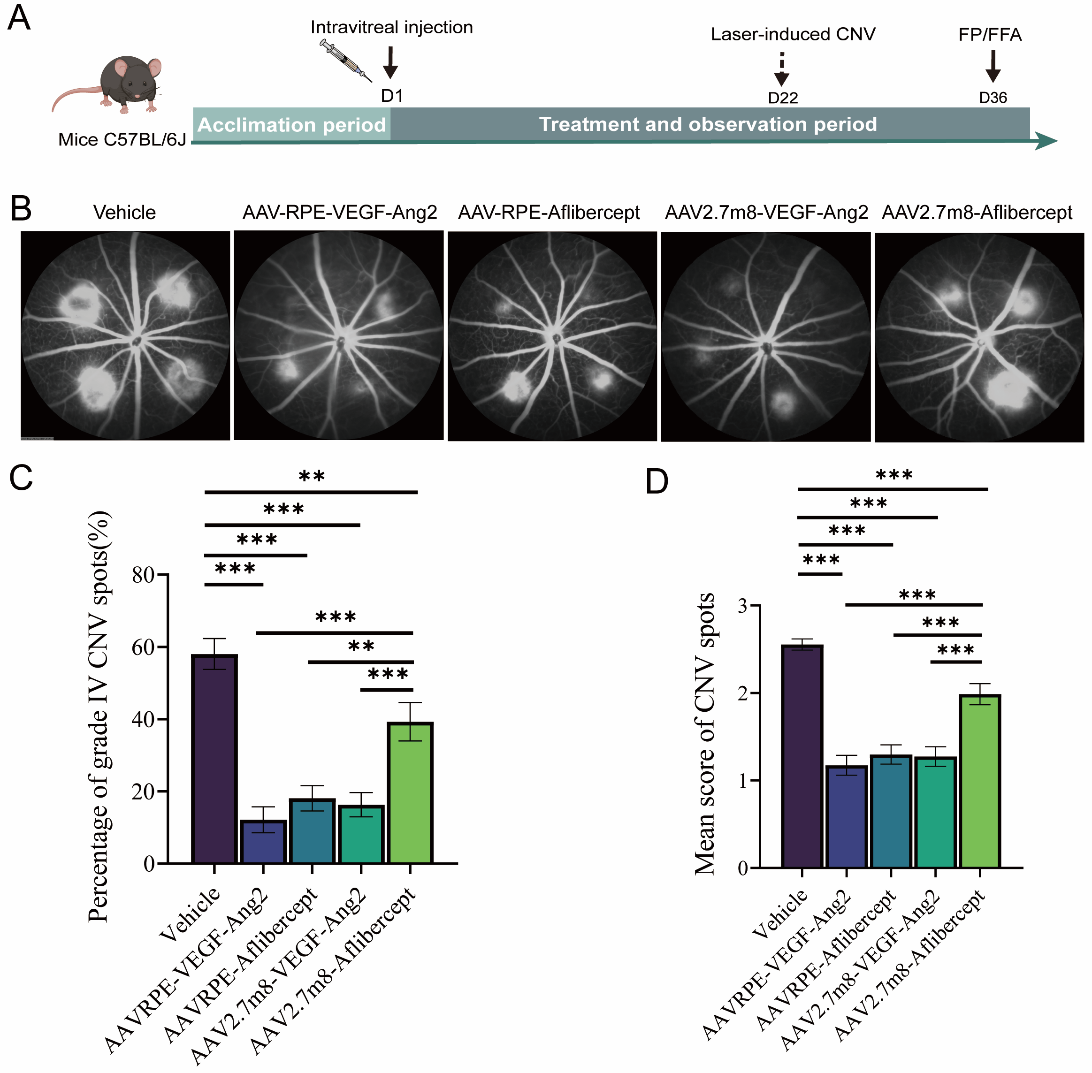
**Fig. S1**

**Fig. S1. Efficacy of AAV-RPE-VEGF-Ang2 and AAV-RPE-Aflibercept on laser-induced CNV lesions in mice.** (A) Schematic diagram of study design. Adult C57BL/6J mice were randomized into five groups (Vehicle ; AAV-RPE-VEGF-Ang2：5E9 vg/eye, AAV-RPE-Aflibercept: 5E9 vg/eye, AAV2.7m8-VEGF-Ang2: 5E9 vg/eye, AAV2.7m8-Aflibercept: 5E9 vg/eye), and were administered via a single intravitreal injection in all eyes. On days 22, the laser photocoagulation was performed in the bilateral fundus of the animals to induce the CNV model. The FFA examinations were performed at days 36, and the efficacy was evaluated based on the percentage of grade IV lesions and the mean score of CNV spots. (B) Representative FFA images showed the CNV spots of all groups. Fluorescence CNV spots gradinge definitions; Grade I: No fluorescence leakage; Grade II: Mild fluorescence leakage, leakage area: 1%–50% of laser spot area; Grade III: Moderate fluorescence leakage, leakage area: 50%–100% of laser spot area; Grade IV: Severe fluorescence leakage, the leakage area is greater than the laser spot size. (C) Changes in percentage of grade IV CNV spots on days 36. (Vehcile: 58.04%±4.28%, AAVRPE-VEGF-Ang2: 12.14%±3.6%, AAVRPE-Aflibercept: 18.1%±3.49%, AAV2.7m8-VEGF-Ang2: 16.3%±3.37%, AAV2.7m8-Aflibercept: 39.29%±5.34%). (D) Changes in mean score of CNV spots on days 36. (Spot ratio of each level (%) = total number of spots of the corresponding grade ÷ total number of spots of four types × 100%). (Vehcile: 2.55±0.06, AAVRPE-VEGF-Ang2: 1.17±0.11, AAVRPE-Aflibercept: 1.30±0.11, AAV2.7m8-VEGF-Ang2: 1.27±0.11, AAV2.7m8-Aflibercept: 1.99±0.12). For the experiment, data are represented as the mean±SEM. Significance is indicated by *P < 0.05, **P < 0.01, and ***P < 0.001 in the figures.

**Fig. S2**


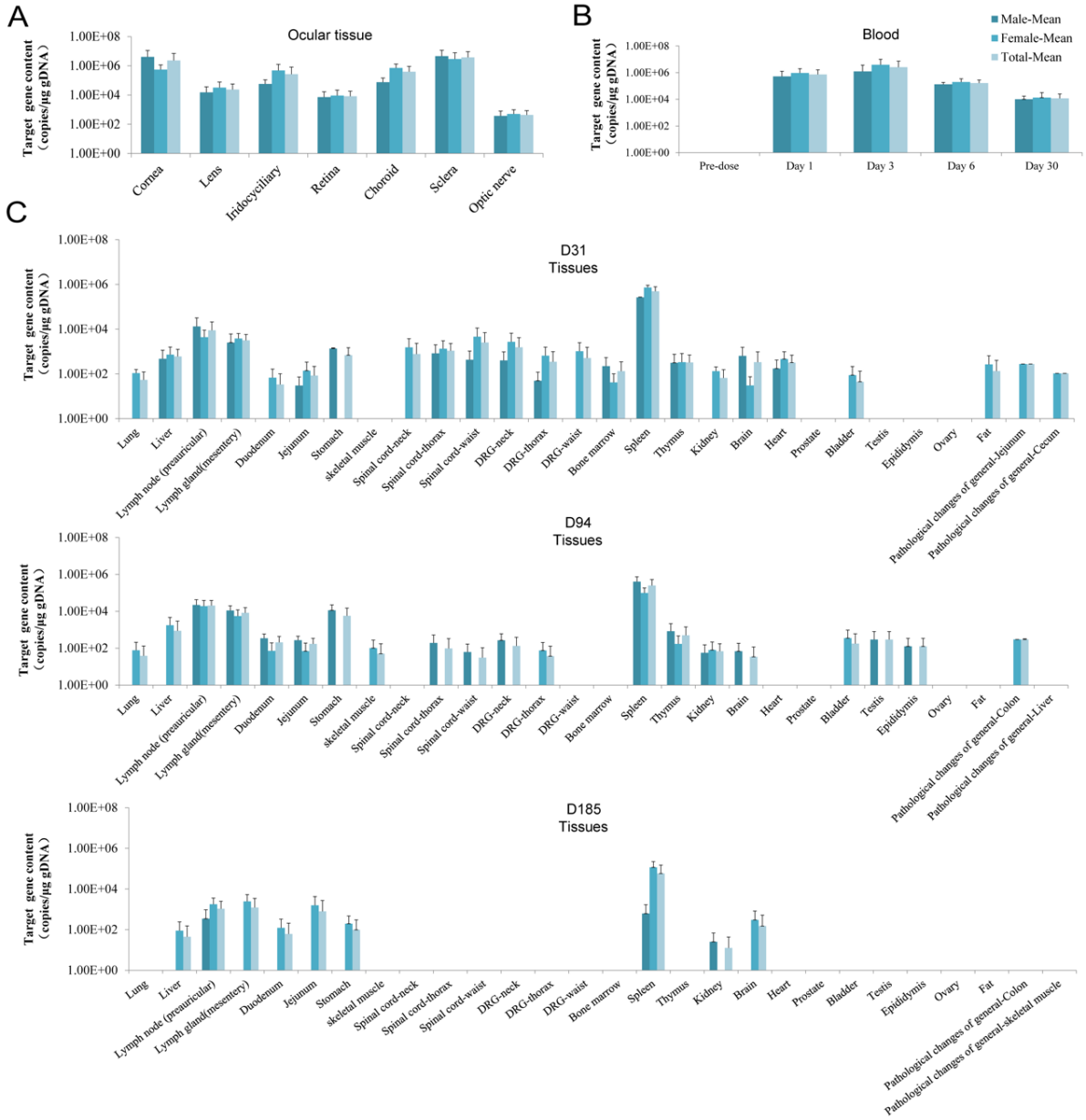


Fig S2. Biodistribution of ocular, blood and tissues of cynomolgus monkeys after intravitreal injection with XMVA09. (A) Mean XMVA09 DNA contents in ocular tissues (OS) on day 185 (n=6 eyes). (B) Mean XMVA09 DNA contents in blood (n=16 animals). (C) Mean XMVA09 DNA contents in tissues after intravitreal injection with XMVA09 (2.5E11 vg/eye) in both eyes of cynomolgus monkeys from day 31 to day 185. Day 31 (n=4 animals), day 94 (n=6 animals), day 185 (n=6 animals). Data were presented as means ± SD, and SD was shown as error bars.

**Fig. S3**


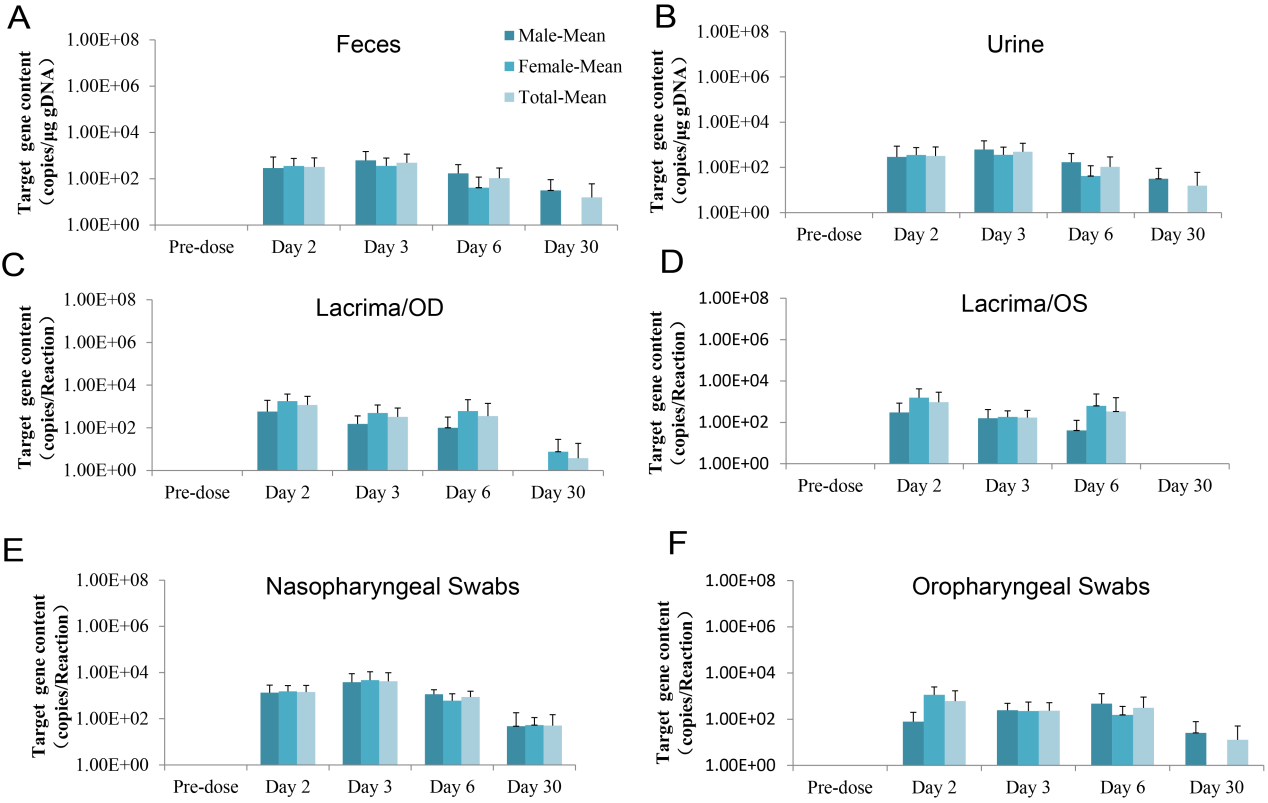


**Fig.S3 Vector shedding in cynomolgus monkeys after intravitreal injection with XMVA09.**

(A and B) Mean XMVA09 DNA contents in feces and urine, XMVA09 (2.5E11 vg/eye, n=16 animals). (C and D) Mean XMVA09 DNA contents in lacrima (n=16 animals). (E and F) Mean XMVA09 DNA contents in nasopharyngeal swabs and oropharyngeal swabs (n=16 animals). Data were presented as means ± SD, and SD was shown as error bars.

**Fig. S4**


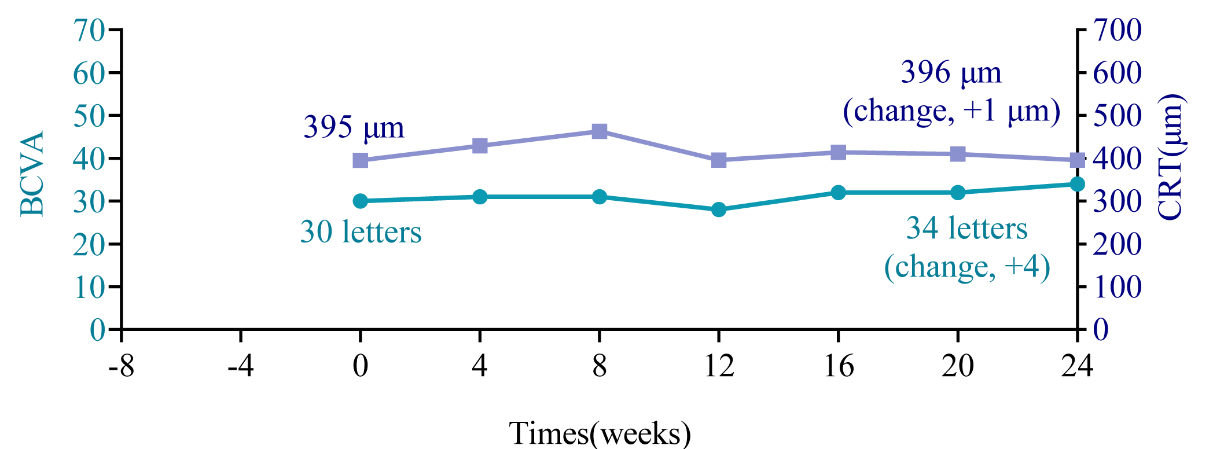


**Fig. S4. Patient 1 was found to have ocular functional changes after treatment.** A 69-year-old woman was diagnosed with "age-related macular degeneration" in her right eye and was treated with XMVA09 (8E10 vg/eye, 100 μL/eye) intravitreal injection. The level of BCVA and CRT changed from baseline within 24 weeks.

**Fig. S5**


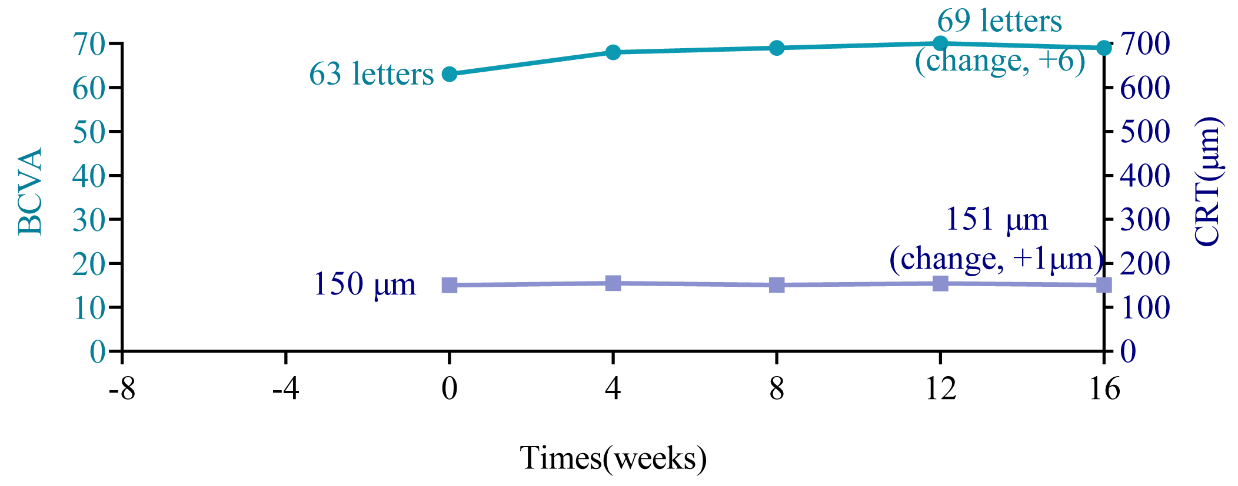


**Fig. S5. Patient 2 was found to have ocular functional changes after treatment.** A 68-year-old woman with decreased binocular vision for about one year and old lesions in the macula of the fundus of her left eye was diagnosed with "exudative age-related macular degeneration in both eyes" and was treated with intravitreal injection of XMVA09 (8E10 vg/eye, 100 μL/eye) in her left eye. Change from baseline in BCVA and CRT levels within 16 weeks.

**Fig. S6**


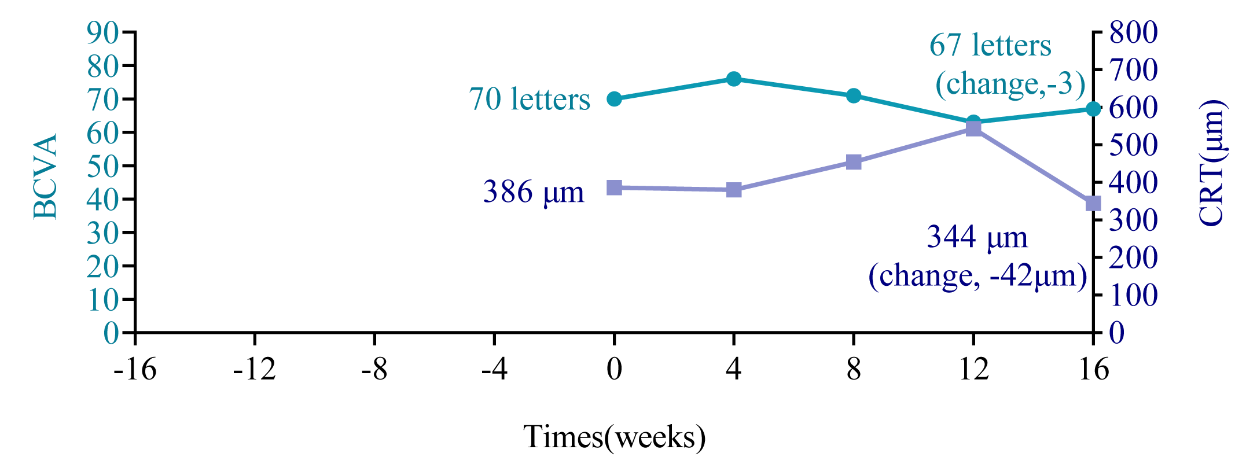


**Fig. S6. Patient 3 was found to have ocular functional changes after treatment.** A 69-year-old man with vision loss in his right eye for about 4 months and slight edema in the fundus of his right eye was diagnosed with "age-related macular degeneration of the right eye" and was treated with XMVA09 (8E10 vg/eye, 100 μL/eye) intravitreal injection. Change from baseline in their BCVA and CRT levels 16weeks was recorded.

**Fig. S7**

**
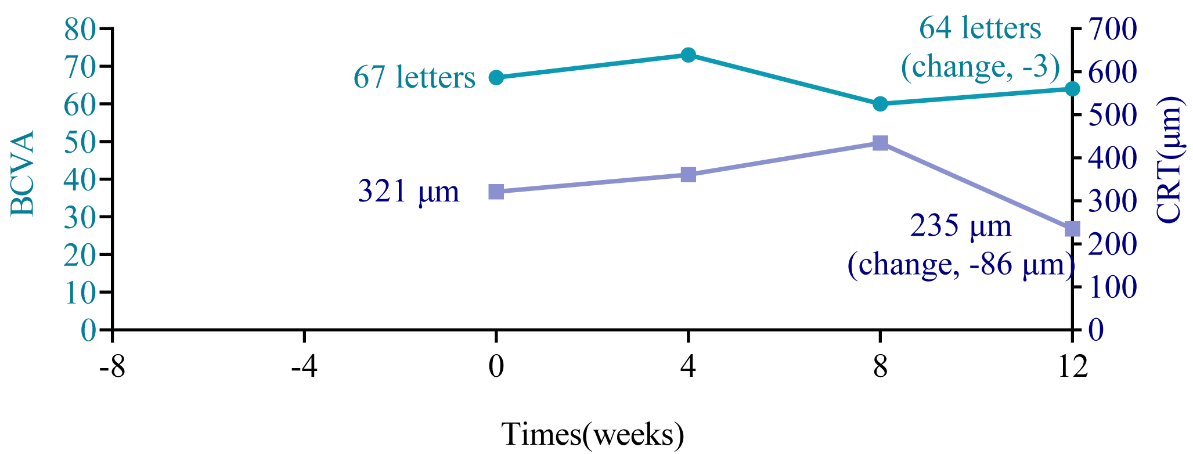
**

**Fig. S7. Patient 4 was found to have ocular functional changes after treatment.** A 69-year-old man with blurred vision in both eyes for one years and an exudative area in the right eye and edema and exudation in the macular area of the left eye was diagnosed with "exudative age-related macular degeneration in both eyes" and was treated with intravitreal injection of XMVA09 (2.50E11 vg/eye, 100 μL/eye). Change from baseline in their BCVA and CRT levels 12 weeks was recorded.

**Fig. S8**


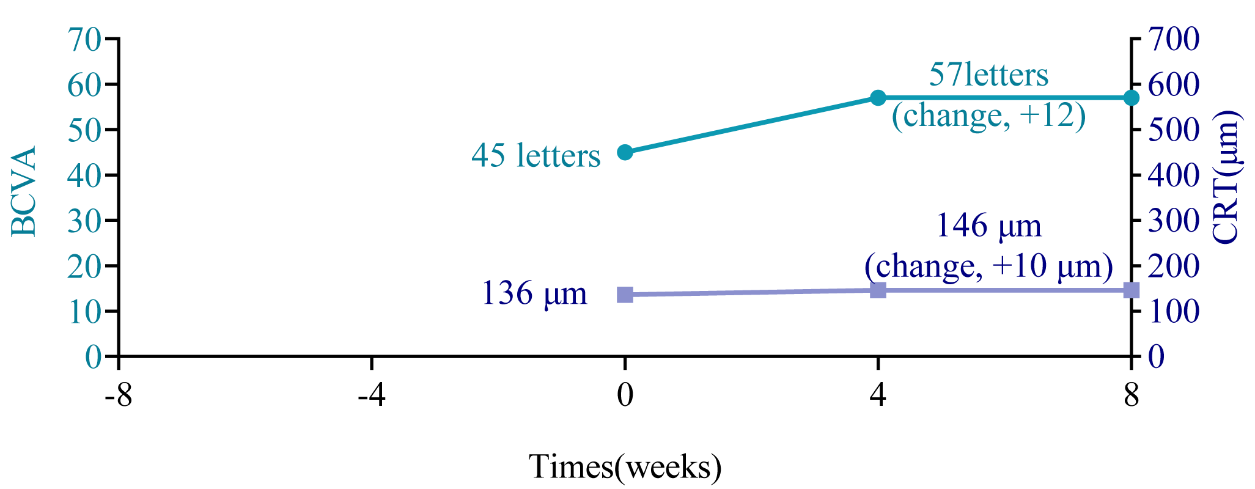


**Fig. S8. Patient 5 was found to have ocular functional changes after treatment.** A 55-year-old man with a grayish-yellow lesion in the macular area of the fundus of the right eye with visual acuity loss in his right eye for half a year was diagnosed with exudative age-related macular degeneration in the right eye and was treated with XMVA09 (2.50E11 vg/eye, 100 μL/eye) intravitreal injection. Changes from baseline in their BCVA and CRT levels 8 weeks were recorded.

**Fig. S9**


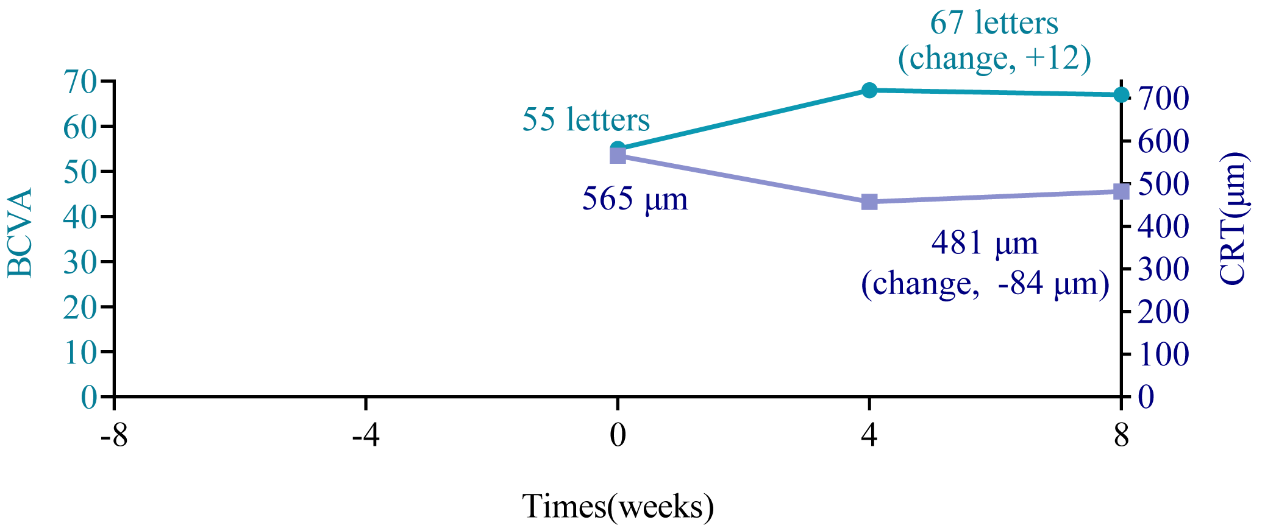


**Fig. S9. Patient 6 was found to have ocular functional changes after treatment.** A 60-year-old man with vision loss in his left eye for about half a year and pigmentation disorder in the macular area of his left eye was diagnosed with "age-related macular degeneration in his left eye". Intravitreal injection of XMVA09 treatment (2.50E11 vg/eye, 100 μL/eye). Change from baseline in BCVA and CRT levels over 8 weeks was recorded.

**Table S1 Grading the Leakage of CNV Lesions in mouse (Based on FFA).**

| **Lesion Grade** | **Definition** |
| --- | --- |
| Grade I | No fluorescence leakage |
| Grade II | Mild fluorescence leakage, the leakage area is 1~50% of the laser spot size |
| Grade III | Moderate fluorescence leakage, the leakage area is 50~100% of the laser spot size |
| Grade IV | Severe fluorescence leakage, the leakage area is greater than the laser spot size |

Table S2 Grading the Leakage of CNV Lesions in rhesus monkeys (Based on FFA).

| **Lesion Grade** | **Definition** |
| --- | --- |
| Grade I | No hyperfluorescence |
| Grade II | Hyperfluorescence without leakage |
| Grade III | Early hyperfluorescence or mid transit and late leakage |
| Grade IV | Transit bright hyperfluorescence with leakage beyond the borders of the burned area |

**Table S3 Analysis of the mean number of grade IV CNV spots** **in rhesus monkeys.**

| Day after Laser  Induction | Day 19 | Day 35 | Day 49 | Day 63 | Day 77 |
| --- | --- | --- | --- | --- | --- |
| Vehicle | 6.17±0.65 | 6.50±0.56 | 5.67±0.71 | 5.67±0.71 | 4.83±0.87 |
| XMVA09 -8.00E10 | 7.17±1.11 | 6.33±1.09 | 5.67±1.31 | 5.17±1.60 | 5.00±1.61 |
| XMVA09  -1.25E11 | 5.63±1.05 | 4.75±0.92 | 3.25±0.88 | 1.88±0.52**^##^ | 1.00±0.33**^##^ |
| XMVA09  -2.50E11 | 4.67±1.59 | 3.17±1.45 | 2.83±1.40 | 2.83±1.47 | 2.33±1.31 |
| XMVA09  -5.00E11 | 1.60±1.12** | 1.00±0.82** | 0.33±0.21** | 0.33±0.21** | 0.33±0.21** |

Data were presented as means ± SEM. Compared with Vehicle group, *p<0.05, **p<0.01. Compared with Day 19, #p<0.05, ##p<0.01.

**Table S4 Analysis of the mean leakage area of grade IV CNV spots in rhesus monkeys.**

| Day after Laser  Induction | Day 19 | Day 35 | Day 49 | Day 63 | Day 77 |
| --- | --- | --- | --- | --- | --- |
| Vehicle | 6.08±0.99 | 7.52±0.96 | 7.53±1.12 | 6.84±1.30 | 5.44±1.45 |
| XMVA09 -8.00E10 | 8.66±2.68 | 8.55±4.30 | 7.47±3.37 | 7.07±2.87 | 7.53±3.09 |
| XMVA09  -1.25E11 | 6.15±1.39 | 3.90±0.60** | 2.51±0.43**^#^ | 1.75±0.34**^##^ | 0.82±0.31**^##^ |
| XMVA09  -2.50E11 | 3.60±1.56 | 1.92±1.05** | 1.92±1.09** | 1.73±0.99* | 1.82±1.10 |
| XMVA09  -5.00E11 | 1.44±1.01** | 0.42±0.28** | 0.33±0.26** | 0.34±0.28** | 0.39±0.32** |

Data were presented as means ± SEM. Compared with Vehicle group, *p<0.05, **p<0.01. Compared with Day 19, #p<0.05, ##p<0.01.

**Table S5 Analysis of the percentage change in retinal thickness of grade IV CNV spots from baseline in rhesus monkeys.**

| Day after Laser  Induction | Day 19 | Day 35 | Day 49 | Day 63 | Day 77 |
| --- | --- | --- | --- | --- | --- |
| Vehicle | 60.72±5.04 | 62.90±3.26 | 62.22±5.81 | 57.70±8.01 | 50.20±8.46 |
| XMVA09 -8.00E10 | 65.99±17.31 | 47.01±12.89 | 49.50±17.98 | 49.11±24.69 | 43.16±21.57 |
| XMVA09  -1.25E11 | 63.29±11.06 | 33.08±3.40**^#^ | 23.29±2.38**^##^ | 18.62±2.30**^##^ | 14.95±2.60**^##^ |
| XMVA09  -2.50E11 | 51.97±12.23 | 23.09±6.15** | 14.76±5.21**^#^ | 9.80±4.06**^##^ | 5.65±2.79**^##^ |
| XMVA09  -5.00E11 | 34.03±15.28 | 10.60±5.86** | 6.14±6.59** | 6.77±8.12** | 5.09±8.64** |

Data were presented as means ± SEM. Compared with Vehicle group, *p<0.05, **p<0.01. Compared with Day 19, #p<0.05, ##p<0.01.

**Table S6 Summary of general ophthalmology examination (male) in cynomolgus monkeys.**

| Abnormal presentation | Group | Dosage (×10^11^  vg/eye) | gender | n | Pre-dose | Day3 | Day8 | Day15 | Day29 | Day59^a^ | Day89^a^ | Day127^b^ | Day183^b^ |
| --- | --- | --- | --- | --- | --- | --- | --- | --- | --- | --- | --- | --- | --- |
| Conjunctival swelling | 1 | Vehicle | ♂ | 16 | 0 | 0 | 0 | 0 | 0 | 0 | 0 | 0 | 0 |
|  | 2 | 1 | ♂ | 10 | 0 | 0 | 0 | 0 | 0 | 0 | 0 | NA | NA |
|  | 3 | 2.5 | ♂ | 16 | 0 | 0 | 0 | 0 | 0 | 0 | 0 | 0 | 0 |
| Conjunctival congestion | 1 | Vehicle | ♂ | 16 | 0 | 0 | 0 | 0 | 0 | 0 | 0 | 0 | 0 |
|  | 2 | 1 | ♂ | 10 | 0 | 0 | 0 | 0 | 0 | 0 | 0 | NA | NA |
|  | 3 | 2.5 | ♂ | 16 | 0 | 3(+) | 0 | 0 | 0 | 0 | 0 | 0 | 0 |
| anterior chamber cells | 1 | Vehicle | ♂ | 16 | 0 | 2(±) | 0 | 0 | 0 | 0 | 0 | 0 | 0 |
|  | 2 | 1 | ♂ | 10 | 0 | 0 | 0 | 0 | 0 | 0 | 0 | NA | NA |
|  | 3 | 2.5 | ♂ | 16 | 0 | 0 | 0 | 0 | 0 | 0 | 0 | 0 | 0 |
| Particle floating | 1 | Vehicle | ♂ | 16 | 0 | 4(±~++) | 5(±~++) | 6(±~+) | 5(±~+) | 3(±) | 3(±) | 1(±) | 1(±) |
|  | 2 | 1 | ♂ | 10 | 0 | 1(±) | 1(+) | 1(++) | 1(++) | 0 | 0 | NA | NA |
|  | 3 | 2.5 | ♂ | 16 | 0 | 2(±~++) | 5(±~+) | 4(±~+) | 3(±~+) | 1(±) | 1(±) | 2(±) | 0 |

Note: “n” indicates number of eyes. “a” indicates that n=12, 6 and 12 in the Vehiclel, XMVA09(1E10 vg/eye) and XMVA09(2.5E11 vg/eye) groups, respectively, on D59 and D89. “b” indicates that n=6 and 6 in the vehicle control and high dose groups, respectively, on D127 and D183. “±, +, ++, +++, ++++” indicate extremely minimal, mild, moderate, marked and severe, respectively. NA indicates no data.

**Table S7 Summary of general ophthalmology examination (female) in cynomolgus monkeys**

| Abnormal presentation | Group | Dosage (×10^11^  vg/eye) | gender | n | Pre-dose | Day3 | Day8 | Day15 | Day29 | Day59^a^ | Day89^a^ | Day127^b^ | Day183^b^ |
| --- | --- | --- | --- | --- | --- | --- | --- | --- | --- | --- | --- | --- | --- |
| Conjunctival swelling | 1 | Vehicle | ♀ | 16 | 0 | 1(+) | 0 | 0 | 0 | 0 | 0 | 0 | 0 |
|  | 2 | 1 | ♀ | 10 | 0 | 0 | 0 | 0 | 0 | 0 | 0 | NA | NA |
|  | 3 | 2.5 | ♀ | 16 | 0 | 0 | 0 | 0 | 0 | 0 | 0 | 0 | 0 |
| Conjunctival congestion | 1 | Vehicle | ♀ | 16 | 0 | 0 | 0 | 0 | 0 | 0 | 0 | 0 | 0 |
|  | 2 | 1 | ♀ | 10 | 0 | 0 | 0 | 0 | 0 | 0 | 0 | NA | NA |
|  | 3 | 2.5 | ♀ | 16 | 0 | 0 | 0 | 0 | 0 | 0 | 0 | 0 | 0 |
| anterior chamber cells | 1 | Vehicle | ♀ | 16 | 0 | 0 | 0 | 0 | 0 | 0 | 0 | 0 | 0 |
|  | 2 | 1 | ♀ | 10 | 0 | 2(±~+) | 0 | 0 | 0 | 0 | 0 | NA | NA |
|  | 3 | 2.5 | ♀ | 16 | 0 | 1(±) | 0 | 0 | 0 | 0 | 0 | 0 | 0 |
| Particle floating | 1 | Vehicle | ♀ | 16 | 0 | 6(±~++) | 6(±~++) | 4(±~+) | 3(±) | 0 | 0 | 1(±) | 0 |
|  | 2 | 1 | ♀ | 10 | 0 | 1(+) | 2(+) | 2(±) | 2(±) | 0 | 0 | NA | NA |
|  | 3 | 2.5 | ♀ | 16 | 0 | 1(±) | 3(+~++) | 1(±) | 6(±~+++) | 1(+++) | 1(+++) | 0 | 0 |

Note: “n” indicates number of eyes. “a” indicates that n=10, 6 and 12 in the Vehiclel, XMVA09(1E10 vg/eye) and XMVA09(2.5E11 vg/eye) groups, respectively, on D59 and D89. “b” indicates that n=4 and 6 in the Vehiclel and XMVA09(2.5E11 vg/eye) groups, respectively, on D127 and D183. “±, +, ++, +++, ++++” indicate minimal, mild, moderate, marked and severe, respectively. The animal #2007 of the vehicle control group was found dead on D52, and thus no relevant data were collected from this animal on D59~183. NA indicates no data.

**Table S8. Analysis of ERG in cynomolgus monkeys.**

| Item | | Vehicle | XMVA09-1E11 vg/eye | XMVA09-2.5E11 vg/eye |
| --- | --- | --- | --- | --- |
| Dark-adapted 10.0 ERG | Scotopic a-wave amplitude(μV) | 150.80±8.47 | 147.40±8.91 | 163.60±5.13 |
|  | Scotopic a-wave peak time(ms) | 12.45±0.33 | 12.08±0.43 | 13.21±0.34 |
|  | Scotopic b-wave amplitude(μV) | 289.50±16.39 | 292.80±23.42 | 295.30±10.26 |
|  | Scotopic b-wave peak time(ms) | 40.41±2.65 | 38.83±1.86 | 38.96±1.58 |
| Dark-adapted 3.0 ERG | Scotopic a-wave amplitude(μV) | 106.40±6.73 | 110.00±7.74 | 119.00±4.68 |
|  | Scotopic a-wave peak time(ms) | 17.59±0.43 | 17.83±0.49 | 17.17±0.40 |
|  | Scotopic b-wave amplitude(μV) | 267.10±15.69 | 276.20±20.89 | 274.80±9.97 |
|  | Scotopic b-wave peak time(ms) | 44.09±3.00 | 42.58±3.34 | 44.63±3.08 |
| Dark-adapted 0.01 ERG | Scotopic a-wave amplitude(μV) | 8.58±1.13 | 9.12±1.20 | 7.74±0.91 |
|  | Scotopic a-wave peak time(ms) | 14.68±1.58 | 14.42±1.18 | 15.38±1.60 |
|  | Scotopic b-wave amplitude(μV) | 145.30±11.76 | 145.20±12.16 | 147.60±6.06 |
|  | Scotopic b-wave peak time(ms) | 103.30±1.84 | 97.75±1.62 | 101.20±2.17 |
| Light-adapted 3.0 ERG | Scotopic a-wave amplitude(μV) | 31.26±1.45 | 29.94±1.65 | 32.50±1.24 |
|  | Scotopic a-wave peak time(ms) | 10.36±0.65 | 10.92±0.89 | 10.00±0.39 |
|  | Scotopic b-wave amplitude(μV) | 112.50±5.74 | 101.20±11.89 | 102.50±4.49 |

**Table S9 Summary of OCT in cynomolgus monkeys.**

| Abnormal presentation | Group | Dosage (×10^11^vg/eye) | Gender | n | Pre-dose | Day29 | Day59^a^ | Day89^a^ | Day183^b^ |
| --- | --- | --- | --- | --- | --- | --- | --- | --- | --- |
| Dot and sheet-like hyperreflective signal was observed in vitreous bodies | 1 | Vehicle | ♂ | 16 | 0 | 0 | 0 | 0 | 0 |
|  | 2 | 1 | ♂ | 10 | 0 | 0 | 0 | 0 | NA |
|  | 3 | 2.5 | ♂ | 16 | 0 | 0 | 0 | 0 | 0 |
|  | 1 | Vehicle | ♀ | 16 | 0 | 2 | 1 | 1 | 0 |
|  | 2 | 1 | ♀ | 10 | 0 | 0 | 1 | 1 | NA |
|  | 3 | 2.5 | ♀ | 16 | 0 | 1 | 1 | 1 | 0 |

Note: "n" indicates number of cases of the administered eye; “a” indicates the number of administered eyes is n=12, in which the number of administered eyes in the female group of group 1 is 10, and the number of administered eyes in the male group and the female group of group 2 is 6; b indicates the number of administered eyes n=6, of which the number of administered eyes in the female group of group 1 is 4; "NA" indicates that there is no relevant data; "♂" for males, "" ♀ for females.

**Table S10 Summary of FP in cynomolgus monkeys.**

|  | Group | Dosage (×10^11^vg/eye) | Gender | n | Pre-dose | Day29 | Day59^a^ | Day89^a^ | Day183^b^ |
| --- | --- | --- | --- | --- | --- | --- | --- | --- | --- |
| Abnormal presentation | 1 | Vehicle | ♂ | 16 | 0 | 0 | 0 | 0 | 0 |
|  | 2 | 1 | ♂ | 10 | 0 | 0 | 0 | 0 | NA |
|  | 3 | 2.5 | ♂ | 16 | 0 | 0 | 0 | 0 | 0 |
|  | 1 | Vehicle | ♀ | 16 | 0 | 0 | 0 | 0 | 0 |
|  | 2 | 1 | ♀ | 10 | 0 | 0 | 0 | 0 | NA |
|  | 3 | 2.5 | ♀ | 16 | 0 | 0 | 0 | 0 | 0 |

Note: "n" indicates the number of cases of the administered eye; “a” indicates that the number of administered eyes is n=12, in which the number of administered eyes in the female group of group 1 is 10, and the number of administered eyes in the male group and the female group of group 2 is 6; b indicates the number of administered eyes n=6, of which the number of administered eyes in the female group of group 1 is 4; "NA" indicates that there is no relevant data; "♂" for males, "" ♀ for females.

**Table S11 Summary of FFA in cynomolgus monkeys.**

|  | Group | Dosage (×10^11^  vg/eye) | Gender | n | Pre-dose | Day29 | Day59^a^ | Day89^a^ | Day183^b^ |
| --- | --- | --- | --- | --- | --- | --- | --- | --- | --- |
| Abnormal presentation | 1 | Vehicle | ♂ | 16 | 0 | 0 | 0 | 0 | 0 |
|  | 2 | 1 | ♂ | 10 | 0 | 0 | 0 | 0 | NA |
|  | 3 | 2.5 | ♂ | 16 | 0 | 0 | 0 | 0 | 0 |
|  | 1 | Vehicle | ♀ | 16 | 0 | 0 | 0 | 0 | 0 |
|  | 2 | 1 | ♀ | 10 | 0 | 0 | 0 | 0 | NA |
|  | 3 | 2.5 | ♀ | 16 | 0 | 0 | 0 | 0 | 0 |

Note: "n" indicates the number of cases of the administered eye; “a” indicates that the number of administered eyes is n=12, in which the number of administered eyes in the female group of group 1 is 10, and the number of administered eyes in the male group and the female group of group 2 is 6; b indicates the number of administered eyes n=6, of which the number of administered eyes in the female group of group 1 is 4; "NA" indicates that there is no relevant data; "♂" for males, "" ♀ for females.

**Table S12 Detection of Anti-AAV Antibody (Serum) in cynomolgus monkeys.**

| Time | Item | Vehicle | XMVA09-  1E11vg/eye | XMVA09-  2.5E11vg/eye |
| --- | --- | --- | --- | --- |
| Pre-dose | Positive /Total Animals | 7/16 | 4/10 | 5/16 |
|  | Titer | 1~9 | 1~81 | 1~81 |
| D23 | Positive /Total Animals | 5/16 | 9/10 | 15/16 |
|  | Titer | 1~9 | 27~729 | 1~2187 |
| D50 | Positive /Total Animals | 6/12 | 5/6 | 10/12 |
|  | Titer | 1~9 | 27~243 | 1~243 |
| D86 | Positive /Total Animals | 4/11 | 6/6 | 8/12 |
|  | Titer | 1~3 | 1~243 | 1~81 |
| D177 | Positive /Total Animals | 0/5 | NA | 3/6 |
|  | Titer | NA | NA | 9~243 |

Note: The animal 2007 in the vehicle control group was found dead on D52, and thus there were no samples collected on D86 or D177.

**Table S13 Detection of Anti-AAV Antibody (Vitreous Humor) in cynomolgus monkeys.**

| Time | Item | Vehicle | XMVA09-1E11  vg/eye | XMVA09-2.5E11  vg/eye |
| --- | --- | --- | --- | --- |
| D31-OD | Positive /Total Animals | 0/4 | 0/4 | 1/4 |
|  | Titer | NA | NA | 3 |
| D31-OS | Positive /Total Animals | 0/4 | 0/4 | 1/4 |
|  | Titer | NA | NA | 3 |
| D94-OD | Positive /Total Animals | 0/6 | 3/6 | 1/6 |
|  | Titer | NA | 9~81 | 3 |
| D94-OS | Positive /Total Animals | 0/6 | 2/6 | 0/6 |
|  | Titer | NA | 1~81 | NA |
| D185-OD | Positive /Total Animals | 0/5 | NA | 1/6 |
|  | Titer | NA | NA | 3 |
| D185-OS | Positive /Total Animals | 0/5 | NA | 1/6 |
|  | Titer | NA | NA | 3 |

Note: Animal 2007 in the vehicle control group was found dead on D52, and thus there were no samples collected on D185. “OD” indicates the oculus dexter, “OS” indicates the oculus sinister.

**Table S14 Summary of Detection Results of Anti-AAV Neutralizing Antibody (Serum) in cynomolgus monkeys.**

| Group | Animal No. | D-14/D-15 | D23 | D50 | D86 | D177 |
| --- | --- | --- | --- | --- | --- | --- |
| Vehicle | 1001 | - | - | NA | NA | NA |
|  | 1002 | - | - | NA | NA | NA |
|  | 1003 | 1 | - | - | - | NA |
|  | 1004 | 9 | 9 | 243 | 9 | NA |
|  | 1005 | - | - | - | - | - |
|  | 1006 | - | - | 3 | 3 | NA |
|  | 1007 | 81 | - | - | - | - |
|  | 1008 | 27 | 3 | 1 | - | - |
|  | 2001 | 27 | 27 | NA | NA | NA |
|  | 2002 | - | - | NA | NA | NA |
|  | 2003 | - | - | - | - | NA |
|  | 2004 | - | 3 | 1 | <1 | NA |
|  | 2005 | 81 | 243 | 100 | 81 | NA |
|  | 2006 | - | - | - | - | - |
|  | 2007 | <1 | - | <1 | ** | ** |
|  | 2008 | - | - | - | - | - |
| XMVA09-1E11  vg/eye | 1101 | 1 | 81 | NA | NA | NA |
|  | 1102 | - | 3 | NA | NA | NA |
|  | 1103 | 9 | - | - | 9 | NA |
|  | 1104 | - | 9 | 27 | 3 | NA |
|  | 1105 | 3 | 300 | 81 | 81 | NA |
|  | 2101 | - | 81 | NA | NA | NA |
|  | 2102 | - | 1 | NA | NA | NA |
|  | 2103 | 9 | 243 | 81 | 81 | NA |
|  | 2104 | - | 27 | 27 | 27 | NA |
|  | 2105 | - | 9 | 243 | 100 | NA |
| XMVA09-2.5E11  vg/eye | 1201 | - | 27 | NA | NA | NA |
|  | 1202 | 27 | 900 | NA | NA | NA |
|  | 1203 | - | 27 | 100 | 81 | NA |
|  | 1204 | 9 | 9 | 9 | 27 | NA |
|  | 1205 | - | 3 | 3 | 1 | NA |
|  | 1206 | - | 27 | 243 | 27 | 27 |
|  | 1207 | - | 3 | - | - | - |
|  | 1208 | - | 1 | 1 | - | - |
|  | 2201 | 9 | 100 | NA | NA | NA |
|  | 2202 | 1 | 81 | NA | NA | NA |
|  | 2203 | - | - | - | - | NA |
|  | 2204 | - | 9 | 9 | 9 | NA |
|  | 2205 | - | 3 | 1 | - | NA |
|  | 2206 | - | 27 | 81 | 81 | 243 |
|  | 2207 | 27 | 100 | 81 | 27 | 27 |
|  | 2208 | - | 3 | 1 | <1 | - |

Note: 1. “**” indicates the animal #2007 was unscheduled dead on D52, so that no samples were collected. “-” indicates no anti-AAV antibody was detected, so that detection of anti-AAV neutralizing antibody was not performed. “NA” indicates not applicable (no samples for detection).

2. The digits indicate the titers of anti-AAV neutralizing antibody in samples.

**Table S15 Summary of Detection Results of Anti-AAV Neutralizing Antibody (Vitreous Humor) in cynomolgus monkeys.**

| Group | Animal No. | D31-OD | D31-OS | D94-OD | D94-OS | D185-OD | D185-OS |
| --- | --- | --- | --- | --- | --- | --- | --- |
| Vehicle | 1001 | - | - | NA | NA | NA | NA |
|  | 1002 | - | - | NA | NA | NA | NA |
|  | 1003 | NA | NA | - | - | NA | NA |
|  | 1004 | NA | NA | - | - | NA | NA |
|  | 1005 | NA | NA | NA | NA | - | - |
|  | 1006 | NA | NA | - | - | NA | NA |
|  | 1007 | NA | NA | NA | NA | - | - |
|  | 1008 | NA | NA | NA | NA | - | - |
|  | 2001 | - | - | NA | NA | NA | NA |
|  | 2002 | - | - | NA | NA | NA | NA |
|  | 2003 | NA | NA | - | - | NA | NA |
|  | 2004 | NA | NA | - | - | NA | NA |
|  | 2005 | NA | NA | - | - | NA | NA |
|  | 2006 | NA | NA | NA | NA | - | - |
|  | 2007 | NA | NA | NA | NA | ** | ** |
|  | 2008 | NA | NA | NA | NA | - | - |
| XMVA09-  1E11vg/eye | 1101 | - | - | NA | NA | NA | NA |
|  | 1102 | - | - | NA | NA | NA | NA |
|  | 1103 | NA | NA | - | - | NA | NA |
|  | 1104 | NA | NA | - | - | NA | NA |
|  | 1105 | NA | NA | 3 | - | NA | NA |
|  | 2101 | - | - | NA | NA | NA | NA |
|  | 2102 | - | - | NA | NA | NA | NA |
|  | 2103 | NA | NA | 3 | 1 | NA | NA |
|  | 2104 | NA | NA | - | - | NA | NA |
|  | 2105 | NA | NA | 81 | 9 | NA | NA |
| XMVA09-  2.5E11vg/eye | 1201 | - | - | NA | NA | NA | NA |
|  | 1202 | <1 | 1 | NA | NA | NA | NA |
|  | 1203 | NA | NA | - | - | NA | NA |
|  | 1204 | NA | NA | 9 | - | NA | NA |
|  | 1205 | NA | NA | - | - | NA | NA |
|  | 1206 | NA | NA | NA | NA | 1 | - |
|  | 1207 | NA | NA | NA | NA | - | - |
|  | 1208 | NA | NA | NA | NA | - | - |
|  | 2201 | - | - | NA | NA | NA | NA |
|  | 2202 | - | - | NA | NA | NA | NA |
|  | 2203 | NA | NA | - | - | NA | NA |
|  | 2204 | NA | NA | - | - | NA | NA |
|  | 2205 | NA | NA | - | - | NA | NA |
|  | 2206 | NA | NA | NA | NA | - | 1 |
|  | 2207 | NA | NA | NA | NA | - | - |
|  | 2208 | NA | NA | NA | NA | - | - |

Note: 1. “**” indicates the animal #2007 was unscheduled dead on D52, so that no samples were collected. “-” indicates no anti-AAV antibody was detected, so that detection of anti-AAV neutralizing antibody testing was not performed. “NA” indicates not applicable (no samples for detection).

2. The digits indicate the titers of anti-AAV neutralizing antibody.

3. OD indicates oculus dextrus (right eye), and OS indicates oculus sinister (left eye).

**Table S16 Summary of Detection Results of Positive Anti-Target Protein Antibody (Serum) in cynomolgus monkeys.**

| Group | Animal No. | Pre-dose | D23 | D50 | D86 | D177 |
| --- | --- | --- | --- | --- | --- | --- |
| Vehicle | 1001 | - | - | NA | NA | NA |
|  | 1002 | - | - | NA | NA | NA |
|  | 1003 | - | - | - | - | NA |
|  | 1004 | - | - | - | - | NA |
|  | 1005 | - | - | - | - | - |
|  | 1006 | - | - | - | - | NA |
|  | 1007 | - | - | - | - | - |
|  | 1008 | - | - | - | - | - |
|  | 2001 | - | - | NA | NA | NA |
|  | 2002 | - | - | NA | NA | NA |
|  | 2003 | - | - | - | - | NA |
|  | 2004 | - | - | - | - | NA |
|  | 2005 | - | - | - | - | NA |
|  | 2006 | - | - | - | - | - |
|  | 2007 | - | - | - | ** | ** |
|  | 2008 | - | - | - | - | - |
| XMVA09-  1E11vg/eye | 1101 | - | - | NA | NA | NA |
|  | 1102 | - | - | NA | NA | NA |
|  | 1103 | - | - | - | - | NA |
|  | 1104 | - | - | - | - | NA |
|  | 1105 | - | - | - | - | NA |
|  | 2101 | - | - | NA | NA | NA |
|  | 2102 | - | - | NA | NA | NA |
|  | 2103 | - | - | - | - | NA |
|  | 2104 | - | - | - | - | NA |
|  | 2105 | - | - | - | - | NA |
| XMVA09-  2.5E11vg/eye | 1201 | - | - | NA | NA | NA |
|  | 1202 | - | - | NA | NA | NA |
|  | 1203 | - | - | - | - | NA |
|  | 1204 | - | - | - | - | NA |
|  | 1205 | - | - | - | - | NA |
|  | 1206 | - | - | - | - | - |
|  | 1207 | - | - | - | - | - |
|  | 1208 | - | - | - | - | - |
|  | 2201 | - | - | NA | NA | NA |
|  | 2202 | - | - | NA | NA | NA |
|  | 2203 | - | - | - | - | NA |
|  | 2204 | - | - | - | - | NA |
|  | 2205 | - | - | - | - | NA |
|  | 2206 | - | - | - | - | - |
|  | 2207 | - | - | - | - | - |
|  | 2208 | - | - | - | - | - |

Note: 1. “-” indicates negative results.

2. “**” indicates the animal 2007 was found dead on D52, so that no samples were collected on D86, D177 and D185.

3. The values were the titers of positive samples. The titer was defined as the maximum dilution factor (except for MRD) at which a sample can generate positive signals.

4. “NA” indicates no sample was available.

**Table S17 Summary of Detection Results of Positive Anti-Target Protein Antibody (Vitreous Humor) in cynomolgus monkeys.**

| Group | Animal No. | D31-OD | D31-OS | D94-OD | D94-OS | D185-OD | D185-OS |
| --- | --- | --- | --- | --- | --- | --- | --- |
| Vehicle | 1001 | - | - | NA | NA | NA | NA |
|  | 1002 | <1 | 1 | NA | NA | NA | NA |
|  | 1003 | NA | NA | - | <1 | NA | NA |
|  | 1004 | NA | NA | - | - | NA | NA |
|  | 1005 | NA | NA | NA | NA | - | - |
|  | 1006 | NA | NA | - | - | NA | NA |
|  | 1007 | NA | NA | NA | NA | - | 1 |
|  | 1008 | NA | NA | NA | NA | - | - |
|  | 2001 | - | - | NA | NA | NA | NA |
|  | 2002 | - | - | NA | NA | NA | NA |
|  | 2003 | NA | NA | - | - | NA | NA |
|  | 2004 | NA | NA | - | - | NA | NA |
|  | 2005 | NA | NA | - | - | NA | NA |
|  | 2006 | NA | NA | NA | NA | - | - |
|  | 2007 | NA | NA | NA | NA | ** | ** |
|  | 2008 | NA | NA | NA | NA | - | - |
| XMVA09-1E11vg/eye | 1101 | - | - | NA | NA | NA | NA |
|  | 1102 | - | - | NA | NA | NA | NA |
|  | 1103 | NA | NA | 1 | - | NA | NA |
|  | 1104 | NA | NA | - | - | NA | NA |
|  | 1105 | NA | NA | - | - | NA | NA |
|  | 2101 | - | - | NA | NA | NA | NA |
|  | 2102 | - | - | NA | NA | NA | NA |
|  | 2103 | NA | NA | - | - | NA | NA |
|  | 2104 | NA | NA | - | - | NA | NA |
|  | 2105 | NA | NA | - | - | NA | NA |
| XMVA09-2.5E11  vg/eye | 1201 | - | - | NA | NA | NA | NA |
|  | 1202 | - | - | NA | NA | NA | NA |
|  | 1203 | NA | NA | <1 | - | NA | NA |
|  | 1204 | NA | NA | - | - | NA | NA |
|  | 1205 | NA | NA | - | - | NA | NA |
|  | 1206 | NA | NA | NA | NA | - | - |
|  | 1207 | NA | NA | NA | NA | - | - |
|  | 1208 | NA | NA | NA | NA | - | - |
|  | 2201 | - | - | NA | NA | NA | NA |
|  | 2202 | - | - | NA | NA | NA | NA |
|  | 2203 | NA | NA | - | - | NA | NA |
|  | 2204 | NA | NA | - | - | NA | NA |
|  | 2205 | NA | NA | - | - | NA | NA |
|  | 2206 | NA | NA | NA | NA | - | - |
|  | 2207 | NA | NA | NA | NA | - | - |
|  | 2208 | NA | NA | NA | NA | - | - |

Note: 1. “-” indicates negative results.

2. “**” indicates the animal 2007 was found dead on D52 and the samples on D86, D177 and D185 were not collected.

3. The values were the titers of positive samples. The titer was defined as the maximum dilution factor (except for MRD) at which a sample can generate positive signals.

4. “OD” indicates the oculus dexter and “OS” indicates the oculus sinister.

5. “NA” indicates no sample was available.

**Table S18 Baseline characteristics of the participants.**

| **Baseline characteristics** | **Cohort1 8E10(n=3)** | **Cohort2 2.5E11(n=3)** | **Total (n=6)** |
| --- | --- | --- | --- |
| Age, years |  |  |  |
| Mean (SD) | 68.70(0.58) | 61.33(7.09) | 65.00(6.03) |
| Sex: n (%) |  |  |  |
| Male | 1(33%) | 3(100%) | 4(67%) |
| Female | 2(67%) | 0(0%) | 2(33%) |
| Race: n (%) |  |  |  |
| Black or African American | 0(0%) | 0(0%) | 0(0%) |
| Asian | 3(100%) | 3(100%) | 6(100%) |
| American Indian or Alaska Native | 0(0%) | 0(0%) | 0(0%) |
| Native Hawaiian or Other Pacific Islander | 0(0%) | 0(0%) | 0(0%) |
| White | 0(0%) | 0(0%) | 0(0%) |
| Multi-Racial | 0(0%) | 0(0%) | 0(0%) |
| Other | 0(0%) | 0(0%) | 0(0%) |
| Baseline BCVA, Letters |  |  |  |
| Mean (SD) | 54.33(21.36) | 55.67(11.02) | 55.00(15.22) |
| Baseline CRT, μm |  |  |  |
| Mean (SD) | 310.33(138.93) | 340.67(215.18) | 325.50(162.84) |

*Data are presented as n (%).

**Table S19. Summary of treatment-emergent adverse events.**

| **Treatment-Emergent Adverse Events** | **Cohort1 8E10**  **(n=3)** | **Cohort2 2.5E11**  **(n=3)** | | **Total**  **(n=6)** |
| --- | --- | --- | --- | --- |
| Serious ocular TEAE, n (%) | 0(0%) | 0(0%) | 0(0%) | |
| Cataract | 0(0%) | 0(0%) | 0(0%) | |
| Retinal detachment | 0(0%) | 0(0%) | 0(0%) | |
| Uveitis | 0(0%) | 0(0%) | 0(0%) | |
| Serious non-ocular TEAE, n (%) |  |  |  | |
| Nonfatal MI | 0(0%) | 0(0%) | 0(0%) | |
| Nonfatal stroke | 0(0%) | 0(0%) | 0(0%) | |
| **High bilirubin** | **0(0%)** | **1(33.33%)** | **1(16.67%)** | |
| Vascular death | 0(0%) | 0(0%) | 0(0%) | |
| Any TEAE of hypertension, n (%) | 0(0%) | 0(0%) | 0(0%) | |
| Ocular TEAEs of Study Participants (Study Eye), n (%) |  |  |  | |
| Anterior Chamber Cell | 0(0%) | 0(0%) | 0(0%) | |
| Vitreal Cells | 0(0%) | 0(0%) | 0(0%) | |
| Anterior Chamber Flare | 0(0%) | 0(0%) | 0(0%) | |
| Conjunctival Haemorrhage | 0(0%) | 0(0%) | 0(0%) | |
| Keratic Precipitates | 0(0%) | 0(0%) | 0(0%) | |
| Vitreous Floaters | 0(0%) | 0(0%) | 0(0%) | |
| Iris Transillumination Defect | 0(0%) | 0(0%) | 0(0%) | |
| Posterior Capsule Opacification | 0(0%) | 0(0%) | 0(0%) | |
| Vitreous Haze | 0(0%) | 0(0%) | 0(0%) | |
| Anterior Chamber Pigmentation | 0(0%) | 0(0%) | 0(0%) | |
| Iris Adhesions | 0(0%) | 0(0%) | 0(0%) | |
| Iris Hyperpigmentation | 0(0%) | 0(0%) | 0(0%) | |
| Lenticular Pigmentation | 0(0%) | 0(0%) | 0(0%) | |
| Cataract | 0(0%) | 0(0%) | 0(0%) | |
| **Dry Eye** | **2(66.67%)** | **0(0%)** | **2(33.33%)** | |
| Iris Atrophy | 0(0%) | 0(0%) | 0(0%) | |
| Punctate Keratitis | 0(0%) | 0(0%) | 0(0%) | |
| Uveitis | 0(0%) | 0(0%) | 0(0%) | |
| Visual Acuity Reduced | 0(0%) | 0(0%) | 0(0%) | |
| Intraocular Pressure Increased | 0(0%) | 0(0%) | 0(0%) | |

*Data are presented as n (%).

**Table S20 Spectral domain optical coherence tomography findings of the patients.**

| **Pt.** | **Age** | **Gender** | **Eye** | **BCVA change from baseline / Baseline(letters)** | **CRT change from baseline/ Baseline(μm)** | **Duration of follow-up(weeks)** |
| --- | --- | --- | --- | --- | --- | --- |
| 1 | 69 | Female | Right | +4（30） | +1（395） | 24 |
| 2 | 68 | Female | Left | +6（63） | +1（150） | 16 |
| 3 | 69 | Male | Right | -3（70） | -42（386） | 16 |
| 4 | 69 | Male | Right | -3（67） | -86（321） | 12 |
| 5 | 55 | Male | Right | +12（45） | +10（136） | 8 |
| 6 | 60 | Male | Left | +12（55） | -84（565） | 8 |

IIT trial Materials and methods

The study eye is defined as the treated eye selected according to the inclusion and exclusion criteria. Only one eye was selected as the study eye, and if both eyes were qualified, the eye with poor vision was selected as the study eye. However, the investigator may choose the eye with better vision based on medical or ethical considerations. From Week 4 to Week 104, participants met any of the following criteria as assessed by the investigator required supplemental anti-VEGF therapy: i) BCVA reduced by ≥10 letters from baseline, and the investigator judged that the visual loss was caused by intraretinal and/or subretinal effusion; ii) CRT increased by ≥75 μm from baseline; iii) severe vision-threatening bleeding caused by AMD; iv) other conditions judged by the investigator to require supplemental treatment.

**Exclusion criteria included**

1.Presence of any retinal or choroidal diseases causing choroidal neovascularization (CNV) other than AMD, or diagnosed polypoidal choroidal vasculopathy (PCV) and deemed unsuitable for inclusion by the investigator; 2.Presence of any other ocular disease affecting central vision other than wAMD, and deemed unsuitable for inclusion by the investigator; 3.Active ocular infection; 4.Uncontrolled glaucoma defined as intraocular pressure >25 mmHg after glaucoma treatment, or severe glaucoma with cup/disc ratio >0.8, or previous glaucoma filtration surgery; 5.Previous or current surgical or laser treatment involving the macula,and deemed unsuitable for inclusion by the investigator; 6.Previous vitrectomy; 7.History of vitreous hemorrhage in the 3 months before screening; 8.Intraocular (periocular) surgery or laser treatment in the 4 weeks before screening; 9.Absence of lens (excluding pseudophakic eyes) or posterior capsular deficiency (excluding eyes with YAG laser posterior capsulotomy after artificial lens implantation); 10.Presence of intraocular implant (excluding intraocular lens) or filler; 11.Intravitreal/intrachoroidal injection of corticosteroids in the 3 months before screening or long-acting intravitreal implant in the 6 months before screening; 12.Clinically significant history of allergy or hypersensitivity to fluorescein sodium and indocyanine green, or allergy to therapeutic or diagnostic proteins, or allergy to two or more drugs and/or non-drug factors, or currently having an allergic disease (except mild allergic diseases considered by researchers ,such as allergic rhinitis, pharyngitis); 13.Uncontrolled hypertension defined as systolic blood pressure ≥160 mmHg or diastolic blood pressure ≥100 mmHg after antihypertensive treatment; 14.Documented history of diabetes mellitus, and deemed unsuitable for inclusion by the investigator; 15.Diagnosed systemic autoimmune diseases or any uncontrolled clinical problem; 16.History of stroke (excluding silent infarcts without clinical symptoms). History of myocardial infarction, cerebrovascular disease, transient ischemic attack, acute coronary syndrome, or coronary revascularization in the 6 months before screening, as well as active disseminated intravascular coagulation and significant bleeding tendency; 17.Surgical history in the 4 weeks before screening ,and deemed unsuitable for inclusion by the investigator; 18.Currently using toxic drugs affecting the lens, retina, or optic nerve; 19.Current systemic infection requiring oral, intramuscular, or intravenous administration; 20.Previous ocular or systemic gene therapy; 21.Screening Period Renal and Hepatic Function Tests: Alanine Aminotransferase (ALT), Aspartate Aminotransferase (AST) above 2 times the upper limit of normal (ULN), or Creatinine (Cr) above 1.5 times the ULN and deemed clinically significant by the investigator; 22.Coagulation dysfunction (prothrombin time ≥3 seconds above the upper limit of the normal range, activated partial thromboplastin time ≥10 seconds above the upper limit of the normal range), deemed unsuitable for inclusion by the investigator; 23.Not using effective contraception (Natural amenorrhea for 12 months;6 weeks after bilateral oophorectomy with/without hysterectomy; Using one or more acceptable contraceptive methods, including, sterilization(male partner bilateral vasectomy, excision), hormonal contraception (implants, patches, oral), intrauterine device, or dual barrier method.;Willing and able to use a reliable contraceptive method throughout the study and comply until the end of the follow-up visits; 24.Pregnant or breastfeeding females; 25.Participation in any systemic medication (excluding vitamins and minerals) clinical trial in the 3 months before screening (or 5 times the half-life if the drug's half-life is longer than 3 months); 26.Deemed unsuitable for inclusion by the investigator; 27.Previous vitrectomy; 28.Absence of lens or posterior capsular deficiency; 29.Presence of intraocular implant (excluding intraocular lens) or filler.

**Any of the following general conditions:**

1.Clinically significant history of allergy or hypersensitivity to fluorescein sodium and indocyanine green, or allergy to therapeutic or diagnostic proteins, or allergy to two or more drugs and/or non-drug factors, or currently having an allergic disease (except mild allergic diseases considered by researchers ,such as allergic rhinitis, pharyngitis); 2.Uncontrolled hypertension defined as systolic blood pressure ≥160 mmHg or diastolic blood pressure ≥100 mmHg after antihypertensive treatment; 3.Documented history of diabetes mellitus, and deemed unsuitable for inclusion by the investigator; 4.Diagnosed systemic autoimmune diseases or any uncontrolled clinical problem; 5.History of stroke (excluding silent infarcts without clinical symptoms). History of myocardial infarction, cerebrovascular disease, transient ischemic attack, acute coronary syndrome, or coronary revascularization in the 6 months before screening, as well as active disseminated intravascular coagulation and significant bleeding tendency.
